# The Genetic Architecture of Murine Glutathione Transferases

**DOI:** 10.1371/journal.pone.0148230

**Published:** 2016-02-01

**Authors:** Lu Lu, Ashutosh K. Pandey, M. Trevor Houseal, Megan K. Mulligan

**Affiliations:** 1 Department of Genetics, Genomics and Informatics, University of Tennessee Health Science Center, Memphis, TN, 38106, United States of America; 2 Co-innovation Center of Neuroregeneration, Nantong University, Nantong, Jiangsu Province, 226001, China; Biogen Idec, UNITED STATES

## Abstract

Glutathione S-transferase (GST) genes play a protective role against oxidative stress and may influence disease risk and drug pharmacokinetics. In this study, massive multiscalar trait profiling across a large population of mice derived from a cross between C57BL/6J (B6) and DBA2/J (D2)—the BXD family—was combined with linkage and bioinformatic analyses to characterize mechanisms controlling GST expression and to identify downstream consequences of this variation. Similar to humans, mice show a wide range in expression of GST family members. Variation in the expression of *Gsta4*, *Gstt2*, *Gstz1*, *Gsto1*, and *Mgst3* is modulated by local expression QTLs (eQTLs) in several tissues. Higher expression of *Gsto1* in brain and liver of BXD strains is strongly associated (*P* < 0.01) with inheritance of the *B6* parental allele whereas higher expression of *Gsta4* and *Mgst3* in brain and liver, and *Gstt2* and *Gstz1* in brain is strongly associated with inheritance of the *D2* parental allele. Allele-specific assays confirmed that expression of *Gsto1*, *Gsta4*, and *Mgst3* are modulated by sequence variants within or near each gene locus. We exploited this endogenous variation to identify coexpression networks and downstream targets in mouse and human. Through a combined systems genetics approach, we provide new insight into the biological role of naturally occurring variants in GST genes.

## Introduction

Cellular life forms, whether autonomous or multicellular, must respond to a constant barrage of environmental and metabolic hazards [1]. Glutathione (GSH) transferase activity evolved as a common strategy to combat threats posed by oxidative stress and xenobiotics [2]. GSH is an important cellular antioxidant, signaling molecule, and cofactor [3]. It is also essential for the maintenance of a suitable redox state in mitochondria—which constantly produce damaging reactive oxygen species as a by-product of cellular respiration. Glutathione S-tranferase (GST) enzymes catalyze the conjugation of GSH to electrophilic compounds [2,4], enabling the subsequent export of these potentially harmful substances out of the cell [5,6]. Thus, GSTs play an important role in detoxification of electrophilic xenobiotics [2,7], including drugs, carcinogens, and pesticides, and in the regulation of key signaling pathways involved in apoptosis, homeostasis, and the cellular stress response [3].

The highly conserved mammalian cytosolic GST superfamily contains over a dozen cytosolic genes divided among 7 classes—alpha, mu, omega, pi, sigma, theta, and zeta [8–10]. Two additional but evolutionarily distinct family members include the soluble Kappa class and the membrane-associated proteins in eicosanoid and glutathione metabolism (MAPEG), which include the microsomal GSTs. All members participate in Phase II detoxification using GSH conjugation. Within many of the classes, additional members were created by duplication events, most occurring before mammalian orders diverged from a common ancestor [11]. As a result, most human genes have homologs in mice and both have additional species-specific paralogs [12,13]. The function of most GST homologs is conserved among mammals with different class members demonstrating specific substrate affinities, although GSTs also have unique roles in diverse biological processes potentially unrelated to detoxification (for a review see [11]).

Many members of the GST superfamily are highly polymorphic among human populations [14] and genetic variation is often associated with differences in enzymatic activity [11]. Conflicting reports of genetic associations between polymorphisms in GST genes and negative outcomes and susceptibility for various cancers or age-related neurodegenerative disorders like Alzheimer’s (AD) and Parkinson’s disease (PD) [15] have been reported in both the overall population and different ethnic groups. This includes polymorphisms in *GSTM1* or *GSTT1* found to be associated with prostate cancer [16,17], acute myeloid leukemia [18], or gastric cancer [19]. In addition, polymorphisms in *GSTP1*, *GSTT1*, *GSTM1*, or *GSTO1* are associated with risk of PD after pesticide exposure [20–23], increase in PD risk [24], AD risk [25,26] or AD age of onset [27–30], respectively. Despite reproducibility issues inherent in most human association and linkage studies, variation in enzyme activity among human populations could lead to differences in drug response, apoptotic cell signaling pathways, or susceptibility to oxidative stress, and further analysis is warranted.

Here we leverage C57BL/6J (B6), DBA/2J (D2), and their recombinant inbred progeny—the BXD family of strains—to investigate the variation and genetic regulation of GST genes. The BXD set has been extensively profiled for thousands of behavioral traits and genome-wide expression levels have been generated for numerous brain regions and peripheral tissues. This population has been densely genotyped and is segregating over five million sequence variants that distinguish the parental lineages. The massive breadth of multiscalar trait data accumulated for the BXD population since the late 1970’s make this family particularly well suited for systems genetics and complex trait analysis. In this study we identify several GST genes with variable expression modulated by local sequence variants—*Gsta4*, *Gstt2*, *Gstz1*, *Mgst3*, and *Gsto1*—and dissect the functional consequences of this variation.

## Methods and Materials

### Ethics statement

All animal work was approved by the Institutional Animal Care and Use Committee (IACUC) at the University of Tennessee Health Science Center (IACUC protocol 12–148.0-B; Legacy protocol 680).

### Generation of BXD recombinant inbred strains

BXD strains were generated from crosses between B6 and D2 strains resulting in a panel of recombinant inbred strains with either the parental *B* or *D* allele fixed at each locus [31]. There are now roughly 160 BXD lines—about 80 strains available from The Jackson Laboratory and ~80 new lines from the University of Tennessee Health Science Center [32]. Animals were maintained on an equal light dark cycle with ad libitum access to food and water.

### GeneNetwork data sets

Detailed information for data sets used in the analysis, including tissue acquisition, RNA extraction, array hybridization methods, data processing, and normalization methods, can be found in the “Info” page at www.genenetwork.org. All mouse expression data sets used in this study were generated through collaborative efforts [33,34] and can be publically accessed from www.genenetwork.org. Representative expression data sets include: VU BXD Midbrain Agilent SurePrint G3 Mouse GE (May12) Quantile [35], Hippocampus Consortium M430v2 (Jun06) [34], VCU BXD PFC Sal M430 2.0 (Dec06) RMA [36], INIA Amygdala Cohort Affy MoGene 1.0 ST (Mar11) RMA, GSE16780 UCLA Hybrid MDP Liver Affy HT M430A (Sep11) [37], and UT-VGX Hepatocytes Affy Mouse Gene 1.0 ST Gene Level (Oct14) RMA. Human cortical expression data sets containing AD and control cases were also used, including GSE15222 Human Brain All Cases Myers (Apr09) RankInv Database [38].

### Gene expression measurements

Expression of cytosolic and membrane-bound GSTs was measured across the BXD family using Affymetrix and Illumina microarray platforms in brain and liver. Representative probe sets (S1 Table) were selected for each gene based on probe set specificity and mean expression. SNPs and other sequence variants, such as indels (insertions and deletions) or CNVs (copy number variants), overlapping probes have an important impact on the false discovery rate of expression differences and on identification of local expression quantitative trait loci (*cis*-eQTLs) [39–41]. No representative probe set target sequences overlap SNPs or other sequence variants that could interfere with GST measurements in this study.

### Heritability calculations

Broad sense heritability [42] was estimated by comparing the genetic variation between strains to the environmental variance within strains. Biological replicates for each strain are required for broad sense heritability calculations and were only performed for expression data sets for which replicates were available. For recombinant inbred lines, which are homozygous at all loci, the following formula was used: 0.5V_A_ / (0.5V_A_ + V_E_) or V_A_ / (V_A_ +2V_E_) where V_A_ = additive genetic variance (variances of strain means) and V_E_ = environmental variance (V_E_ = V_T_—V_A_, V_T_ = total variance among subjects) **[43]**.

### QTL mapping

Genome-wide interval mapping of gene expression patterns across the BXDs using different data sets was performed in GeneNetwork (www.genenetwork.org), as previously described [44]. Genome-wide empirical P-values are estimated by permuting trait data for each transcript randomly 5000 or more times, to determine the genome-wide significance threshold (P value < 0.05). We estimated QTL intervals for downstream analysis based on a 1.5 LOD confidence interval.

### Analysis of allele-specific expression by RNA-seq

We downloaded paired-end RNA-seq data from the European Nucleotide Archive (accession number ERP000591) for lung, liver, and hippocampus of B6xD2 F1 female hybrids generated by crossing B6 females with D2 males [45]. The data consist of transcriptome sequence from six biological replicates. We aligned RNA-seq reads to both the B6 reference genome (mm10 assembly) and the SNP-substituted D2 genome using “Splice Transcripts Alignment to a Reference” tool (STAR, version 2.3.1a) [46] with the following parameters: “—outFilterMultimapNmax 10—outFilterMismatchNmax 12”. We used SAMtools (version 0.1.19) “pileup” function [47] and an in-house Python script (https://github.com/ashutoshkpandey/ASE_prealignment/blob/master/Allele_specific_SAM.py) to assign reads overlapping SNPs to either the B6 or D2 parental allele using both parental genomes. We calculated allelic ratios for each SNP as the ratio of number of reads assigned to the reference allele (B6) to the total number of aligned reads (B6+D2). For each SNP we used an interquartile range (IQR) method to identify outlier allelic ratios from the set of six F1 replicates. Outlier ratios were located outside the [Q1–1.5(IQR) and Q3 + 1.5(IQR)] range where Q1 and Q3 represent first and third quartiles and IQR is calculated as Q3 –Q1. Reads from replicates showing concordant allelic ratios were merged and allelic ratios were recalculated. We used the chi-square goodness of fit test to determine allelic imbalances for a given SNP. For a SNP showing an allelic imbalance, the ratio will deviate from 0.5. We defined genes as having an allele-specific expression difference if they contained one or more SNPs with an allelic imbalance at an FDR threshold of less than 0.1 [48]. We also required the expression fold difference to be >1.25.

### Global protein quantification of *Gsto1*

Data from global protein quantification of mouse hippocampus using tandem mass tag technology was mined for *Gsto1* peptides. Hippocampal tissue was collected from three B6 and three D2 mice of two different ages (2 to 3 months or 12 months of age). Isobaric labeling was used to differentiate sample peptides and 10 fractions were collected for each sample and analyzed by low pH reverse phase LC-MS/MS. A total of 7,074 proteins were identified in this study, of which 7,014 were quantified. 28 spectral counts and 8 peptides were identified for *Gsto1*. A t-test (p < 0.05) was used to determine significance.

### Partial correlation analysis

Pearson product-moment correlations were computed in GeneNetwork between *Gsto1* (probe set 1416531_at) and all other probe sets in the Hippocampus Consortium M430v2.0 data set after controlling for the genetic variation near *Gsto1* (locus rs13483647; Chr 19 at 46.66 Mb) and *Ina* (locus rs13483649; Chr 19 at 47.22 Mb). The residual values after partial correlation for *Gsto1* are stored in the BXD Published Phenotypes database as trait ID 17328. One hundred forty highly correlated and relevant genes were selected based on the following criteria: partial correlation *P* < 0.001 (*r* ≥ |0.38|) and a literature correlation of *r* > 0.5. Literature correlations are available on GeneNetwork and were originally computed using the Semantic Gene Organizer (SGO) software to automatically extract gene-gene relations from titles and abstracts in MEDLINE citations [49]. The Chilibot system (http://chilibot.net), Alzheimer Disease & Frontotemporal Dementia Mutation Database (http://www.molgen.ua.ac.be/ADMutations/), and the ALZGENE database (www.alzgene.org) were used to scan candidate genes for relations with AD. Results were also checked manually.

### Identification of downstream targets and phenotypes

The following analytical steps were taken to identify transcripts that are putative downstream targets. First, transcripts with expression level greater than 8 units modulated by a suggestive *trans*-*e*QTL (LOD > 2) located in a 4 Mb region overlapping the location of the target GST gene in at least one tissue were selected. Second, the correlation coefficient between the target GST and the expression of each *trans*-regulated gene was computed (*p* < 0.01 is considered a significant correlation).

To identify phenotypes that are likely to be downstream of the variation in GST genes we queried a phenotype database of nearly 5000 published phenotypes to identify those significantly correlated with the expression of each GST gene. We also identified phenotypes whose expression was significantly regulated by genomic variants situated within 2 Mb of each target GST gene locus.

## Results

### Variation in expression of several GST family members is under genetic control

Genome-wide expression profiles are available for ~20 peripheral tissues or cell types and ~10 individual brain regions for the BXD population (www.genenetwork.org). We surveyed all available data but chose to focus on several representative data sets from brain (hippocampus, prefrontal cortex, amygdala, and midbrain) and peripheral tissue (liver and liver hepatocytes) for detailed systems genetics analysis. Representative data sets are robust for expression QTL mapping (eQTL), have been extensively error-checked, and include a large number of BXD strains.

GST expression levels vary tremendously across B6, D2, and the BXD population both within and across tissues (Table 1, S1 Table). Variation tends to be greater in liver compared to individual brain regions. Some GSTs, such as *Gsta2* and *Gsta3*, are predominately expressed in liver, whereas others have higher expression in brain, such as *Gstm5* and *Mgst3*. The expression of many GSTs is moderately to highly heritable (H^2^ > 0.3) suggesting that some of the variation in expression of individual GSTs is controlled by genetic factors. In agreement with this observation, several GSTs demonstrate genetic modulation of expression by local (cis eQTLs) or distant (trans eQTLs) loci (S1 Table). In general, distant eQTLs are not well conserved across tissues but cis eQTLs are often highly reproducible. We surveyed the genetic regulation of GST family members from all seven classes and found consistent cis modulation of expression across multiple tissues for *Gsta4*, *Gstt2*, *Gstz1*, *Gsto1*, and *Mgst3* (Table 2). No significant and consistent cis or trans modulation of expression was detected for mu, pi, or kappa class members or for the sigma class, which is represented by the highly specialized *Hpgds* gene that converts prostaglandin H2 to prostaglandin D2. With the exception of *Gsto1*, all cis-modulated genes (*Gsta4*, *Gstt2*, *Gstz1*, and *Mgst3*) have higher expression in the BXD population that is associated with inheritance of the *D* allele from the D2 parental strain. For all cis-modulated GST family members we leveraged multiple informatics resources to identify the genetic architecture underlying expression variation, explore potential functional consequences of that variation, and evaluate biological function through coexpression analysis.

**Table 1:** Summary of GST gene expression.

Symbol	Human Homolog	Chr	Mb	Agilent Probe Set	Mid Mean	Mid FC	Mid LOD	Mid QTL	Affymetrix Probe Set	Hip Mean	Hip FC	Hip H^2	Hip LOD	Hip QTL	Liv Mean	Liv FC	Liv LOD	Liv QTL
**Class Alpha**																		
*Gsta1*	NA	9	78.08	A_55_P2032946	*5*.*95*	1.38	**3.7**	trans	-	-	-		-	-	-	-	-	-
*Gsta2*	*GSTA5*	9	78.15	A_55_P2170454	*6*.*24*	2.91	**8.9**	cis	1421040_a_at	*5*.*69*	1.51	0.28	1.9	trans	11.14	8.07	**3.1**	trans
*Gsta3*	*GSTA3*	1	21.25	A_55_P1961423	9.02	1.24	**3.7**	trans	1423436_at	*5*.*76*	1.36	0.24	1.9	trans	13.16	3.46	2.8	trans
*Gsta4*	GSTA4	9	78.05	A_51_P112223	10.36	1.23	**10.5**	cis	1416368_at	10.88	1.99	**0.59**	**14.2**	cis	11.33	11.43	4.1	cis
**Class Mu**																		
*Gstm1*	*GSTM5*	3	107.82	A_55_P1966432	11.45	1.19	**3.1**	trans	1416416_x_at	12.12	1.81	**0.34**	1.9	trans	14.50	1.83	2.3	trans
*Gstm2*	*GSTM1*	3	107.78	A_55_P1966438	7.91	1.55	**3.3**	trans	1416411_at	7.15	1.85	**0.43**	2.4	trans	10.15	3.21	2.7	trans
*Gstm3*	NA	3	107.77	A_55_P2065231	10.15	1.37	2.7	trans	1427474_s_at	9.22	1.69	**0.33**	2.6	trans	10.63	16.11	2.9	trans
*Gstm4*	*GSTM4*	3	107.84	A_51_P327585	9.28	1.17	**3.1**	trans	1424835_at	8.38	1.33	0.26	1.7	trans	10.09	1.97	2.6	trans
*Gstm5*	*GSTM3*	3	107.70	A_51_P260169	11.86	1.17	1.9	trans	1416842_at	13.14	1.34	0.29	2.2	trans	9.15	2.02	2.9	trans
*Gstm6*	NA	3	107.74	A_55_P2031676	9.15	1.41	2.4	trans	1422072_a_at	8.22	1.51	**0.32**	2.6	trans	9.55	4.44	2.4	trans
*Gstm7*	*GSTM2*	3	107.73	A_55_P2016667	10.03	1.17	**3.1**	trans	1419072_at	9.74	1.61	**0.30**	2.5	trans	10.31	2.10	2.5	trans
**Class Pi**																		
*Gstp1*	*GSTP1*	19	4.04	A_51_P374464	11.70	1.14	**3.9**	trans	*-*	-	-	-	-	-	-	-	-	-
*Gstp2*	*GSTP1*	19	4.04	A_55_P2008704	11.80	1.15	2.2	trans	1449575_a_at	13.57	1.28	**0.30**	2.5	trans	15.48	1.42	**3.3**	trans
**Class Theta**																		
*Gstt1*	*GSTT1*	10	75.25	A_55_P2024841	10.84316216	1.182631	2.2	trans	1418186_at	8.96	1.59	**0.39**	2.5	trans	13.28	2.59	2.4	trans
*Gstt2*	*GSTT2*	10	75.29	A_51_P350048	9.453054054	1.273677475	**5.0**	cis	1417883_at	7.10	1.65	**0.39**	**4.1**	cis	12.71	1.72	2.4	trans
*Gstt3*	NA	10	75.24	A_51_P377856	10.11583784	1.235418637	**3.3**	trans	1423891_at	*5*.*50*	1.46	**0.49**	2.1	trans	9.94	7.72	2.9	trans
Gstt4	NA	10	75.28	A_51_P432229	*5*.*98527027*	1.680627504	2.1	trans	-	-	-	-	-	-	-	-		
**Class Sigma**																		
*Hpgds*	*HPGDS*	*2*	25.32	A_52_P536796	8.03	1.44	**3.1**	trans	1423859_a_at	*6*.*46*	1.44	**0.55**	**2.1**	trans	*6*.*37*	1.22	**2.8**	trans
**Class Zeta**																		
*Gstz1*	*GSTZ1*	12	88.50	A_55_P1988708	11.18	1.27	**17.4**	cis	1427552_a_at	8.57	1.56	0.24	2.9	cis	13.10	1.38	**4.5**	trans
**Class Omega**																		
*Gsto1*	*GSTO1*	19	47.94	A_51_P155313	11.23	1.28	**16.4**	cis	1416531_at	10.61	1.63	**0.69**	**18.8**	cis	12.41	1.80	**4.3**	cis
Gsto2	*GSTO2*	19	47.95	A_55_P2019233	8.96	1.24	2.5	trans	1453708_a_at	*5*.*49*	1.36	**0.39**	2.6	trans	-	-		
**Class Kappa**																		
*Gstk1*	*GSTK1*	6	42.20	A_55_P2051313	10.46	1.19	2.6	trans	1452823_at	8.75	1.66	**0.339114752**	2.4	trans	12.45	1.68	2.7	trans
**MAPEG Family**																		
*Mgst1*	*MGST1*	6	138.10	A_55_P2175880	10.39	1.23	1.9	trans	1415897_a_at	9.07	2.76	**0.49**	**3.2**	trans	14.93	1.21	1.8	trans
*Mgst2*	*MGST2*	3	51.49	A_51_P150120	8.74	1.65	1.7	trans	1452592_at	*6*.*43*	1.26	0.24	3.0	trans	*6*.*65*	1.27	1.9	trans
*Mgst3*	*MGST3*	1	169.30	A_55_P2056342	11.62	1.53	**18.2**	cis	1448300_at	11.61	3.21	**0.56**	**17.7**	cis	9.27	7.43	**10.3**	cis

Chr = chromosome, Mb = megabase, Mid = midbrain, Hip = hippocampus, Liv = liver, FC = fold change, H^2 = broad sense heritability.

**Table 2:** Summary of cis modulation of expression.

Group	Dataset	Tissue	Record	Gene	Position	Mean Tissue Expr	Max LRS	Max LOD	QTL Peak Marker Location	Additive Effect
BXD	EPFL/LISP BXD CD Brown Adipose Affy Mouse Gene 2.0 ST Gene Level (Oct13) RMA	Adipose	17519649	*Gsta4*	Chr9: 78.04	11.91	30.9	6.70	Chr9: 77.25	0.31
BXD	INIA Adrenal Affy MoGene 1.0ST (Jun12) RMA	Adrenal Gland	10587315	*Gsta4*	Chr9: 78.04	10.74	25.7	5.57	Chr9: 77.25	0.25
BXD	SJUT Cerebellum mRNA M430 (Oct04) MAS5	Cerebellum	1416368_at_A	*Gsta4*	Chr9: 78.05	9.36	10.4	2.26	Chr9: 77.22	0.19
BXD	Hippocampus Consortium M430v2 (Jun06) RMA	Hippocampus	1416368_at	*Gsta4*	Chr9: 78.05	10.91	65.5	14.21	Chr9: 77.25	0.19
BXD	UTHSC Hippocampus Illumina v6.1 All Combined (Nov12) RankInv	Hippocampus	ILM1660369	*Gsta4*	Chr9: 78.06	13.16	56.4	12.23	Chr9: 78.05	0.54
BXD	EPFL/LISP BXD CD+HFD Liver Affy Mouse Gene 1.0 ST (Apr13) RMA	Liver	10587315	*Gsta4*	Chr9: 78.04	11.25	20	4.34	Chr9: 78.05	0.21
BXD	GSE16780 UCLA Hybrid MDP Liver Affy HT M430A (Sep11) RMA	Liver	1416368_at_A	*Gsta4*	Chr9: 78.05	11.43	18.9	4.10	Chr9: 77.22	0.48
BXD	VU BXD Midbrain Agilent SurePrint G3 Mouse GE (May12) Quantile	Midbrain	A_51_P112223	*Gsta4*	Chr9: 78.05	10.36	48.5	10.52	Chr9: 77.22	0.08
BXD	EPFL/LISP BXD CD+HFD Muscle Affy Mouse Gene 1.0 ST (Dec11) RMA	Muscle	10587315	*Gsta4*	Chr9: 78.04	9.75	16.3	3.54	Chr9: 78.05	0.13
BXD	HQF BXD Neocortex ILM6v1.1 (Dec10v2) RankInv	Neocortex	ILM1660369	*Gsta4*	Chr9: 78.06	13.13	25.6	5.55	Chr9: 78.05	0.15
BXD	INIA Pituitary Affy MoGene 1.0ST (Jun12) RMA	Pituitary Gland	10587315	*Gsta4*	Chr9: 78.04	9.53	19.4	4.21	Chr9: 77.25	0.17
BHF2	UCLA BHF2 Brain Female mlratio	Brain	10024394148	*Gsta4*	Chr9: 78.04	0.00	15.9	3.45	Chr9: 76.10	0.02
BHF2	UCLA BHF2 Liver Male mlratio	Liver	10024394148	*Gsta4*	Chr9: 78.04	-0.03	33.3	7.22	Chr9: 76.10	0.07
BXD	Eye M430v2 (Sep08) RMA	Eye	1417883_at	*Gstt2*	Chr10: 75.29	9.16	39.1	8.48	Chr10: 74.44	0.17
BXD	UTHSC Mouse BXD Gastrointestinal Affy MoGene 1.0 ST Gene Level (Apr14) RMA	Gastrointestinal Tract	10370013	*Gstt2*	Chr10: 75.29	9.34	25.3	5.49	Chr10: 74.08	0.11
BXD	Hippocampus Consortium M430v2 (Jun06) RMA	Hippocampus	1417883_at	*Gstt2*	Chr10: 75.29	7.10	18.8	4.08	Chr10: 73.39	0.08
BXD	UTHSC Hippocampus Illumina v6.1 NOS (Sep09) RankInv	Hippocampus	ILM1580519	*Gstt2*	Chr10: 75.29	7.57	14.3	3.10	Chr10: 74.08	0.09
BXD	Mouse kidney M430v2 Female (Aug06) RMA	Kidney	1417883_at	*Gstt2*	Chr10: 75.29	12.79	18.1	3.93	Chr10: 71.42	0.19
BXD	HZI Lung M430v2 (Apr08) RMA	Lung	1417883_at	*Gstt2*	Chr10: 75.29	9.97	30.1	6.53	Chr10: 74.08	0.14
BXD	VU BXD Midbrain Agilent SurePrint G3 Mouse GE (May12) Quantile	Midbrain	A_51_P350048	*Gstt2*	Chr10: 75.29	9.45	23.2	5.03	Chr10: 74.44	0.06
BXD	HQF BXD Neocortex ILM6v1.1 (Dec10v2) RankInv	Neocortex	ILM1580519	*Gstt2*	Chr10: 75.29	7.85	11.3	2.45	Chr10: 74.08	0.06
BXD	Hippocampus Consortium M430v2 (Jun06) RMA	Hippocampus	1427552_a_at	*Gstz1*	Chr12: 88.50	8.55	13.5	2.93	Chr12: 86.99	0.06
BXD	VU BXD Midbrain Agilent SurePrint G3 Mouse GE (May12) Quantile	Midbrain	A_55_P1988708	*Gstz1*	Chr12: 88.50	11.18	80.2	17.40	Chr12: 88.25	0.10
BXD	VCU BXD NAc Sal M430 2.0 (Oct07) RMA	Nucleus Accumbens	1427552_a_at	*Gstz1*	Chr12: 88.50	9.16	16.5	3.58	Chr12: 85.87	0.08
BXD	VCU BXD PFC EtOH M430 2.0 (Dec06) RMA	Prefrontal Cortex	1427552_a_at	*Gstz1*	Chr12: 88.50	7.17	21.1	4.58	Chr12: 87.45	0.11
AXBXA	IRCM AXB/BXA Mouse Heart ILM MouseRef-8 v2.0 (Feb13) RankInv	Heart	ILMN_1229964	*Gstz1*	Chr12: 88.51	8.22	23.7	5.14	Chr12: 87.28	-0.06
BHF2	UCLA BHF2 Brain (June05) mlratio	Brain	10024414602	*Gstz1*	Chr12: 88.49	0.01	69.7	15.12	Chr12: 88.36	0.03
CTB6F2	UCLA CTB6/B6CTF2 Brain Males (2005) mlratio	Brain	10018188926	*Gstz1*	Chr12: 88.49	-0.04	17.6	3.82	Chr12: 86.97	-0.02
CTB6F2	UCLA CTB6B6CTF2 Liver Female mlratio	Liver	10018188926	*Gstz1*	Chr12: 88.49	-0.02	91.5	19.85	Chr12: 85.35	-0.11
CTB6F2	UCLA CTB6B6CTF2 Muscle Male mlratio	Muscle	10018188926	*Gstz1*	Chr12: 88.49	-0.03	59.1	12.82	Chr12: 86.97	-0.10
BXD	INIA Amygdala Cohort Affy MoGene 1.0 ST (Mar11) RMA	Amygdala	10463836	*Gsto1*	Chr19: 47.93	9.74	48.2	10.46	Chr19: 46.66	0.17
BXD	UCHSC BXD Whole Brain M430 2.0 (Nov06) RMA	Brain	1416531_at	*Gsto1*	Chr19: 47.94	10.16	35.1	7.61	Chr19: 48.45	-0.13
BXD	Eye M430v2 (Sep08) RMA	Eye	1416531_at	*Gsto1*	Chr19: 47.94	14.14	15.1	3.28	Chr19: 47.22	-0.17
BXD	GNF Stem Cells U74Av2 (Mar04) RMA	Hematopoietic Cells	97819_at	*Gsto1*	Chr19: 47.94	9.36	16.5	3.58	Chr19: 47.22	-0.16
BXD	UMCG Progenitor Cells ILM6v1.1 (Apr09) original	Hematopoietic Cells	ILM6650600	*Gsto1*	Chr19: 47.94	12.22	22.4	4.86	Chr19: 47.22	-0.25
BXD	Hippocampus Consortium M430v2 (Jun06) RMA	Hippocampus	1416531_at	*Gsto1*	Chr19: 47.94	10.63	86.7	18.81	Chr19: 47.22	-0.17
BXD	UTHSC Hippocampus Illumina v6.1 All Combined (Nov12) RankInv	Hippocampus	ILM6650600	*Gsto1*	Chr19: 47.94	11.62	34.7	7.53	Chr19: 46.67	-0.31
BXD	INIA Hypothalamus Affy MoGene 1.0 ST (Nov10)	Hypothalamus	10463836	*Gsto1*	Chr19: 47.94	9.64	19.2	4.16	Chr19: 47.22	0.11
BXD	Mouse Kidney M430v2 (Jul06) RMA	Kidney	1416531_at	*Gsto1*	Chr19: 47.94	11.96	29.1	6.31	Chr19: 47.22	-0.34
BXD	GSE16780 UCLA Hybrid MDP Liver Affy HT M430A (Sep11) RMA	Liver	1416531_at_A	*Gsto1*	Chr19: 47.94	12.45	19.7	4.27	Chr19: 47.22	-0.13
BXD	HZI Lung M430v2 (Apr08) RMA	Lung	1416531_at	*Gsto1*	Chr19: 47.94	12.07	44.5	9.65	Chr19: 46.66	-0.26
BXD	VU BXD Midbrain Agilent SurePrint G3 Mouse GE (May12) Quantile	Midbrain	A_51_P155313	*Gsto1*	Chr19: 47.94	11.23	75.6	16.40	Chr19: 47.22	-0.10
BXD	EPFL/LISP BXD CD+HFD Muscle Affy Mouse Gene 1.0 ST (Dec11) RMA	Muscle	10463836	*Gsto1*	Chr19: 47.93	9.79	25.5	5.53	Chr19: 46.66	-0.14
BXD	BIDMC/UTHSC Dev Neocortex P14 ILMv6.2 (Nov11) RankInv	Neocortex	ILMN_1254523	*Gsto1*	Chr19: 47.94	12.09	27.9	6.05	Chr19: 48.08	-0.20
BXD	VCU BXD NAc Sal M430 2.0 (Oct07) RMA	Nucleus Accumbens	1416531_at	*Gsto1*	Chr19: 47.94	10.39	29	6.29	Chr19: 48.45	-0.08
BXD	INIA Pituitary Affy MoGene 1.0ST (Jun12) RMA	Pituitary Gland	10463836	*Gsto1*	Chr19: 47.93	7.81	62.2	13.49	Chr19: 47.22	-0.29
BXD	VCU BXD PFC EtOH M430 2.0 (Dec06) RMA	Prefrontal Cortex	1416531_at	*Gsto1*	Chr19: 47.94	8.73	33.1	7.18	Chr19: 47.22	-0.14
BXD	VCU BXD PFC Sal M430 2.0 (Dec06) RMA	Prefrontal Cortex	1416531_at	*Gsto1*	Chr19: 47.94	8.75	34.3	7.44	Chr19: 47.22	-0.16
BXD	Normal HEI Retina (April 2010) RankInv	Retina	ILMN_1254523	*Gsto1*	Chr19: 47.94	12.03	21.7	4.71	Chr19: 47.22	-0.38
BXD	IoP Affy MOE 430v2 Spleen (May09) RMA	Spleen	1416531_at	*Gsto1*	Chr19: 47.94	10.78	27.9	6.05	Chr19: 47.22	-0.22
BXD	UTHSC Affy MoGene 1.0 ST Spleen (Dec10) RMA	Spleen	10463836	*Gsto1*	Chr19: 47.93	9.66	76.5	16.59	Chr19: 47.22	-0.27
BXD	UTK Spleen ILM6.1 (Jan10) VST	Spleen	ILM6650600	*Gsto1*	Chr19: 47.94	13.01	56.1	12.17	Chr19: 47.22	-0.59
BXD	HQF Striatum Affy Mouse Exon 1.0ST Gene Level (Dec09) RMA	Striatum	6870181	*Gsto1*	Chr19: 47.93	9.48	14.4	3.12	Chr19: 48.45	-0.18
BXD	HZI Treg M430v2 (Feb11) RMA	T Cell (regulatory)	1416531_at	*Gsto1*	Chr19: 47.94	10.71	41.6	9.02	Chr19: 47.22	-0.37
BXD	INIA Brain mRNA M430 (Jan06) RMA	Brain	1416531_at_A	*Gsto1*	Chr19: 47.94	10.57	27.4	5.94	Chr19: 47.22	-0.10
BXD	UNC Agilent G4121A Liver LOWESS Stanford (Jan06) Both Sexes	Liver	A_51_P155313	*Gsto1*	Chr19: 47.94	0.03	47.7	10.35	Chr19: 46.66	-0.30
AXBXA	GSE16780 UCLA Mouse AXB/BXA Liver Affy HT M430A (Sep11) RMA	Liver	1416531_at_A	*Gsto1*	Chr19: 47.94	12.51	36	7.81	Chr19: 46.66	-0.18
AXBXA	GSE16780 UCLA Mouse AXB/BXA Liver Affy HT M430A (Sep11) RMA	Liver	1456036_x_at_A	*Gsto1*	Chr19: 47.94	10.58	41.9	9.09	Chr19: 46.66	-0.25
LXS	Hippocampus Illumina NOE (Oct08) RankInv beta	Hippocampus	ILM6650600	*Gsto1*	Chr19: 47.94	12.34	18.1	3.93	Chr19: 48.45	0.20
MDP	UCLA GSE27483 MDP Bone Femur ILM Mouse WG-6 v1, v1.1 (Jan13) RSN	Bone Femur	ILM6650600	*Gsto1*	Chr19: 47.94	12.46	22.6	4.90	Chr19: 48.21	-0.68
BXD	EPFL/LISP BXD CD Brown Adipose Affy Mouse Gene 2.0 ST Gene Level (Oct13) RMA	Adipose	17229403	*Mgst3*	Chr1: 169.30	9.85	21.8	4.73	Chr1: 169.15	0.19
BXD	INIA Amygdala Cohort Affy MoGene 1.0 ST (Mar11) RMA	Amygdala	10359861	*Mgst3*	Chr1: 169.30	9.66	23	4.99	Chr1: 169.15	0.11
BXD	UCHSC BXD Whole Brain M430 2.0 (Nov06) RMA	Brain	1448300_at	*Mgst3*	Chr1: 169.30	11.30	31.4	6.81	Chr1: 168.32	0.22
BXD	Eye M430v2 (Sep08) RMA	Eye	1448300_at	*Mgst3*	Chr1: 169.30	11.23	22.8	4.95	Chr1: 169.15	0.20
BXD	UTHSC Mouse BXD Gastrointestinal Affy MoGene 1.0 ST Gene Level (Apr14) RMA	Gastrointestinal Tract	10359861	*Mgst3*	Chr1: 169.30	11.76	27.9	6.05	Chr1: 168.32	-0.13
BXD	Hippocampus Consortium M430v2 (Jun06) RMA	Hippocampus	1448300_at	*Mgst3*	Chr1: 169.30	11.48	81.6	17.70	Chr1: 169.15	0.35
BXD	UTHSC Hippocampus Illumina v6.1 All Combined (Nov12) RankInv	Hippocampus	ILM3450338	*Mgst3*	Chr1: 169.30	14.17	75.8	16.44	Chr1: 169.15	0.94
BXD	UTHSC Hippocampus Illumina v6.1 All Combined (Nov12) RankInv	Hippocampus	ILM5290736	*Mgst3*	Chr1: 169.30	11.86	85.6	18.57	Chr1: 169.15	0.85
BXD	INIA Hypothalamus Affy MoGene 1.0 ST (Nov10) Female	Hypothalamus	10359861	*Mgst3*	Chr1: 169.30	8.95	13.2	2.86	Chr1: 169.15	0.11
BXD	Mouse Kidney M430v2 (Jul06) RMA	Kidney	1448300_at	*Mgst3*	Chr1: 169.30	13.35	57	12.36	Chr1: 169.15	0.85
BXD	EPFL/LISP BXD CD+HFD Liver Affy Mouse Gene 1.0 ST (Apr13) RMA	Liver	10359861	*Mgst3*	Chr1: 169.30	9.68	58.9	12.78	Chr1: 169.15	0.43
BXD	GSE16780 UCLA Hybrid MDP Liver Affy HT M430A (Sep11) RMA	Liver	1448300_at_A	*Mgst3*	Chr1: 169.30	8.97	47.3	10.26	Chr1: 168.32	0.81
BXD	SUH BXD Liver CCl4-treated Affy Mouse Gene 1.0 ST (Jun11) RMA	Liver	10359861	*Mgst3*	Chr1: 169.30	9.82	30.9	6.70	Chr1: 169.15	0.33
BXD	VU BXD Midbrain Agilent SurePrint G3 Mouse GE (May12) Quantile	Midbrain	A_55_P2056342	*Mgst3*	Chr1: 169.30	11.62	83.9	18.20	Chr1: 169.15	0.19
BXD	EPFL/LISP BXD CD+HFD Muscle Affy Mouse Gene 1.0 ST (Dec11) RMA	Muscle	10359861	*Mgst3*	Chr1: 169.30	11.56	46.6	10.11	Chr1: 169.15	0.16
BXD	BIDMC/UTHSC Dev Neocortex P14 ILMv6.2 (Nov11) RankInv	Neocortex	ILMN_1238479	*Mgst3*	Chr1: 169.30	13.16	53.7	11.65	Chr1: 169.15	0.33
BXD	HQF BXD Neocortex ILM6v1.1 (Dec10v2) RankInv	Neocortex	ILM3450338	*Mgst3*	Chr1: 169.30	14.89	59	12.80	Chr1: 169.15	0.23
BXD	HQF BXD Neocortex ILM6v1.1 (Dec10v2) RankInv	Neocortex	ILM5290736	*Mgst3*	Chr1: 169.30	13.46	66.4	14.40	Chr1: 169.15	0.31
BXD	VCU BXD NAc Sal M430 2.0 (Oct07) RMA	Nucleus Accumbens	1448300_at	*Mgst3*	Chr1: 169.30	11.77	47.4	10.28	Chr1: 169.15	0.30
BXD	INIA Pituitary Affy MoGene 1.0ST (Jun12) RMA	Pituitary Gland	10359861	*Mgst3*	Chr1: 169.30	7.60	33.7	7.31	Chr1: 169.15	-0.13
BXD	Full HEI Retina (April 2010) RankInv	Retina	ILMN_1238479	*Mgst3*	Chr1: 169.30	10.52	46.2	10.02	Chr1: 169.15	0.22
BXD	UTHSC Affy MoGene 1.0 ST Spleen (Dec10) RMA	Spleen	10359861	*Mgst3*	Chr1: 169.30	9.72	15.9	3.45	Chr1: 169.15	0.29
BXD	HBP Rosen Striatum M430V2 (Apr05) RMA Orig	Striatum	1448300_at	*Mgst3*	Chr1: 169.30	10.78	18.7	4.06	Chr1: 168.32	0.36
BXD	HQF Striatum Affy Mouse Exon 1.0ST Gene Level (Dec09) RMA	Striatum	6763903	*Mgst3*	Chr1: 169.30	10.47	12.4	2.69	Chr1: 168.32	0.15
BXD	HQF BXD Striatum ILM6.1 (Dec10v2) RankInv	Striatum	ILM3450338	*Mgst3*	Chr1: 169.30	13.51	15.6	3.38	Chr1: 168.91	0.15
BXD	HQF BXD Striatum ILM6.1 (Dec10v2) RankInv	Striatum	ILM5290736	*Mgst3*	Chr1: 169.30	12.16	26.2	5.68	Chr1: 168.91	0.19
BXD	INIA Brain mRNA M430 (Jun06) RMA	Brain	1448300_at_A	*Mgst3*	Chr1: 169.30	12.40	37.6	8.16	Chr1: 168.32	0.32
BXD	SJUT Cerebellum mRNA M430 (Mar05) RMA	Cerebellum	1448300_at_A	*Mgst3*	Chr1: 169.30	12.46	55.7	12.08	Chr1: 168.91	0.48
BXD	GE-NIAAA Cerebellum mRNA M430v2 (May05) RMA	Cerebellum	1448300_at	*Mgst3*	Chr1: 169.30	12.18	28.4	6.16	Chr1: 169.15	0.57
BXD	UNC Agilent G4121A Liver LOWESS Stanford (Jan06) Both Sexes	Liver	A_51_P215077	*Mgst3*	Chr1: 169.31	0.10	71	15.40	Chr1: 168.91	0.58
B6D2F2	OHSU/VA B6D2F2 Brain mRNA M430 (Aug05) RMA	Brain	1448300_at_A	*Mgst3*	Chr1: 169.30	11.41	63.7	13.82	Chr1: 168.63	0.35
CTB6F2	UCLA CTB6/B6CTF2 Brain (2005) mlratio	Brain	10024412567	*Mgst3*	Chr1: 169.30	-0.04	323.4	70.15	Chr1: 169.30	0.13
CTB6F2	UCLA CTB6/B6CTF2 Liver (2005) mlratio	Liver	10024412567	*Mgst3*	Chr1: 169.30	-0.05	268	58.13	Chr1: 169.30	0.26
CTB6F2	UCLA CTB6/B6CTF2 Muscle (2005) mlratio	Muscle	10024412567	*Mgst3*	Chr1: 169.30	-0.05	167.3	36.29	Chr1: 169.30	0.08

Abbreviations as follows: Expr = Expression; BXD = recombinant inbred cross between C57BL/6 (B) and DBA/2 (D); AXBXA = recombinant inbred set derived from reciprocal crosses between A/J (A) and C57BL/6J (B); BHF2 = F2 cross between C57BL/6J (B) and C3H/HeJ (H); CTB6F2 = F2 cross between CAST/EiJ (CT) and C57BL/6 (B6); LXS = recombinant inbred set generated from Inbred Long-Sleep (ILS) and Short-Sleep (ISS) mice that originated from a cross between 8 inbred strains; MDP = Mouse Diversity Panel consisting of 122 inbred strains of mice; B6D2F2 = F2 cross between C57BL/6 (B6) and DBA/2 (D2). In general a positive additive effect means higher expression of the gene is associated with inheritance of the parental allele and a negative additive effect indicates higher expression driven by inheritance of the maternal allele. Crosses are written with the maternal strain first, such that the maternal strain of the BXD set is C57BL/6 (B).

### Gsta4

**Fig 1:**
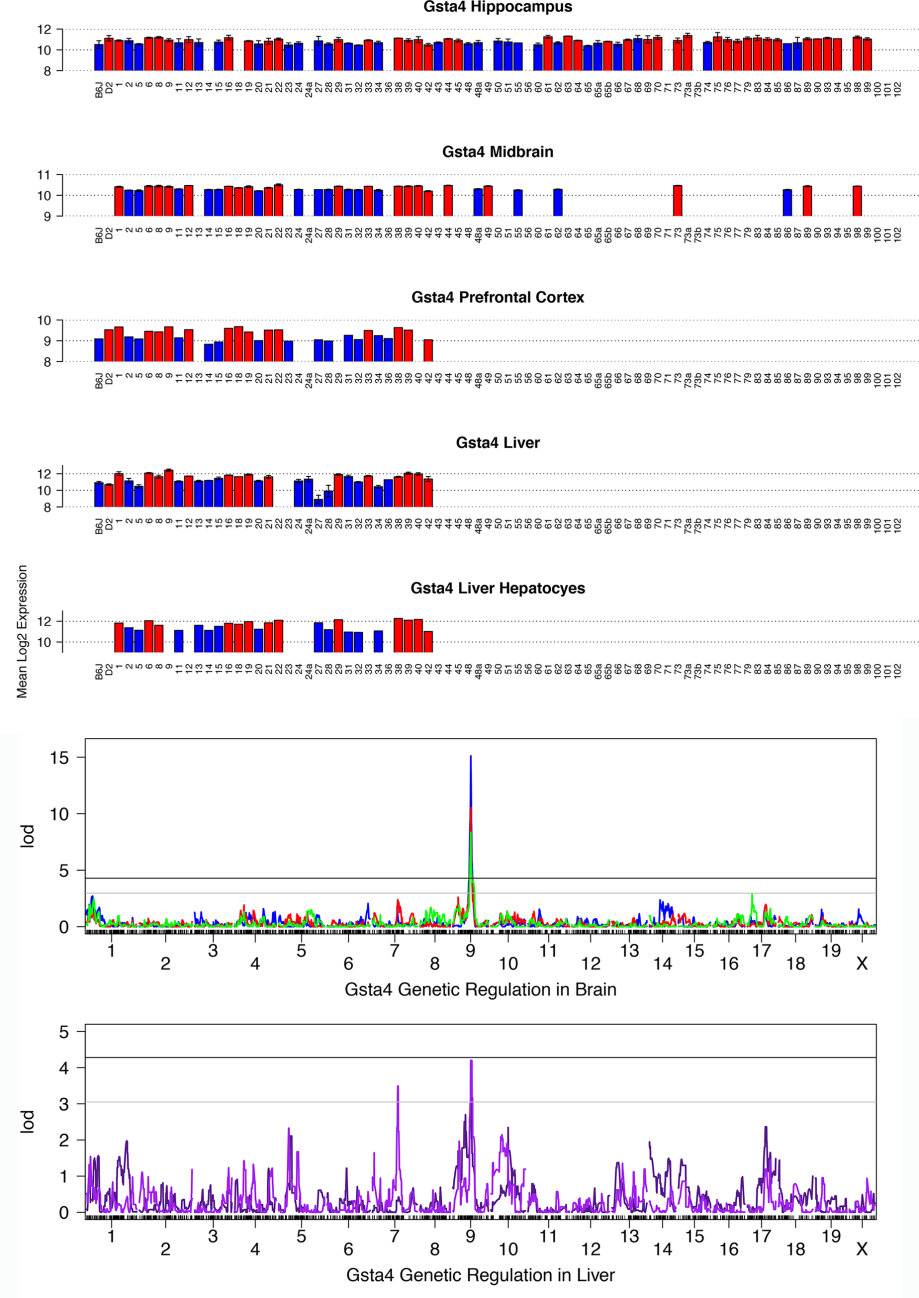
Summary of expression and QTL mapping across BXD strains for *Gsta4*. The top five panels contain bar plots representing the expression of *Gsta4* for each BXD individual in five different tissues. Average log_2_ expression is shown on the y-axis and unique strains are shown on the X-axis (BXD1 = 1). Red and blue indicate inheritance of the paternal D2 (*D*) or maternal B6 (*B***)** allele of *Gsta4* in each strain, respectively. If only a single individual was used for expression measurements, error bars are not shown. For genetic reference populations, mapping power is derived from the number of individuals as opposed to the number of biological replicates. Higher expression is associated with inheritance of the *D* allele. Bottom two panels show the genetic mapping results in each tissue. Association strength (LOD) is shown on the Y-axis and plotted across the genome on the X-axis (by chromosome) for hippocampus (blue), midbrain (red), prefrontal cortex (green), liver (dark purple), and hepatocytes (purple). Genome-wide significance is determined by permutation (*n* = 5000) with the threshold for significance indicated as black (significant, *p* <0.05) and grey (suggestive, *p* < 0.3) horizontal lines. Expression of *Gsta4* is modulated by variants within or near its own locus on Chr 9, a cis eQTL.

Expression is variable across the BXD set (Fig 1) ranging from a modest level in the midbrain (fold change of 1.23) to high levels in prefrontal cortex, hippocampus, and liver (fold changes of 1.99, 1.80, and 11.43, respectively). This variation is highly heritable (H^2^ = 0.59) and is strongly modulated by a significant cis eQTL in 10 tissues (Table 2), including midbrain (LOD = 10.5), hippocampus (LOD = 14.2), prefrontal cortex (LOD = 7.5), liver (LOD = 4), and liver hepatocytes (LOD = 4.2) (Fig 1). The 1.5 LOD confidence interval, which defines the boundaries of the QTL, is ~3 Mb (77 to 80 Mb on Chr 9), precisely overlapping the location of the gene (Chr 9 at 78 Mb). Higher expression of *Gsta4* in the BXD family is driven by inheritance of the *D* allele (Fig 1). Expression of *Gsta4* is also cis-modulated in an F2 intercross between C57BL/6J and C3H/HeJ (BHF2, Table 2) and higher expression is driven by inheritance of the C3H/HeJ allele. This result is consistent with a mutation within or near *Gsta4* that occurred in the B6 parental strain.

*Gsta4* is situated within a relatively variant-rich region on Chr 9; 96 SNPs and 22 small insertion/deletions (indels) are segregating between the B6 and D2 parental strains. None of these variants are in coding exons, known regulatory regions, or splice sites. Instead, one or more of these variants alter expression through undefined regulatory regions or alternative mechanisms. Although the causal variant has not yet been identified, the cis eQTL regulating *Gsta4* expression in multiple tissues across the BXD panel has been validated in liver, lung, and hippocampus by allele-specific expression (ASE) assays using RNA sequencing in B6xD2 F1 hybrid mice (Table 3).

**Table 3:** Summary of GST ASE.

Gene Symbol	Chromosome	Position	B6	D2	B6 counts	D2 counts	Total reads	P-value	Allelic ratio	RefSeq transcript	Genic feature	Tissue
*Gsta4*	chr9	78192047	C	A	317	699	1016	0.00E+00	0.31	NM_010357	5' UTR	Liver
*Gsta4*	chr9	78192047	C	A	72	165	237	0.00E+00	0.30	NM_010357	5' UTR	Lung
*Gsta4*	chr9	78192047	C	A	86	161	247	1.82E-06	0.35	NM_010357	5' UTR	Hip
*Gstz1*	chr12	87164437	C	T	3902	4007	7909	2.38E-01	0.49	NM_010363	3' UTR	Liver
*Gstz1*	chr12	87164437	C	T	139	153	292	4.13E-01	0.48	NM_010363	3' UTR	Lung
*Gstz1*	chr12	87164437	C	T	96	117	213	1.50E-01	0.45	NM_010363	3' UTR	Hip
*Gsto1*	chr19	47855077	G	A	281	208	489	9.63E-04	0.57	NM_010362	5' UTR	Liver
*Gsto1*	chr19	47857874	T	C	511	393	904	8.69E-05	0.57	NM_010362	Exon 3	Liver
*Gsto1*	chr19	47858048	A	G	464	300	764	3.02E-09	0.61	NM_010362	Exon 3	Liver
*Gsto1*	chr19	47864318	G	A	1098	720	1818	0.00E+00	0.60	NM_010362	Exon 6	Liver
*Gsto1*	chr19	47864511	G	A	466	270	736	0.00E+00	0.63	NM_010362	3' UTR	Liver
*Gsto1*	chr19	47864609	T	C	694	421	1115	0.00E+00	0.62	NM_010362	3' UTR	Liver
*Gsto1*	chr19	47855077	G	A	255	173	428	7.38E-05	0.60	NM_010362	5' UTR	Lung
*Gsto1*	chr19	47857874	T	C	145	90	235	3.33E-04	0.62	NM_010362	Exon 3	Lung
*Gsto1*	chr19	47858048	A	G	161	92	253	1.44E-05	0.64	NM_010362	Exon 3	Lung
*Gsto1*	chr19	47864318	G	A	264	227	491	9.50E-02	0.54	NM_010362	Exon 6	Lung
*Gsto1*	chr19	47864511	G	A	228	149	377	4.73E-05	0.60	NM_010362	3' UTR	Lung
*Gsto1*	chr19	47864609	T	C	224	140	364	1.07E-05	0.62	NM_010362	3' UTR	Lung
*Gsto1*	chr19	47855077	G	A	63	48	111	1.55E-01	0.57	NM_010362	5' UTR	Hip
*Gsto1*	chr19	47857874	T	C	90	71	161	1.34E-01	0.56	NM_010362	Exon 3	Hip
*Gsto1*	chr19	47858048	A	G	88	74	162	2.71E-01	0.54	NM_010362	Exon 3	Hip
*Gsto1*	chr19	47864318	G	A	142	104	246	1.54E-02	0.58	NM_010362	Exon 6	Hip
*Gsto1*	chr19	47864511	G	A	112	76	188	8.65E-03	0.60	NM_010362	3' UTR	Hip
*Gsto1*	chr19	47864609	T	C	114	85	199	3.98E-02	0.57	NM_010362	3' UTR	Hip
*Mgst3*	chr1	167372500	A	G	374	865	1239	0.00E+00	0.30	NM_025569	3' UTR	Liver
*Mgst3*	chr1	167372500	A	G	105	108	213	8.37E-01	0.49	NM_025569	3' UTR	Lung
*Mgst3*	chr1	167372500	A	G	257	307	564	3.53E-02	0.46	NM_025569	3’ UTR	Hip

Hip = Hippocampus.

#### Multiple phenotypes are modulated by variation at the *Gsta4* locus

Traits (e.g. gene expression or behavioral phenotypes) that map back to the physical location of *Gsta4* may be modulated in part by variation in *Gsta4* expression. A repository of behavioral, metabolic, and pharmacological traits (The BXD Published Phenotypes Database) was queried to find all phenotypes that mapped within 4 Mb of the eQTL confidence interval (Chr 9 from 73 to 83 Mb) with a LOD score of two or better. Measures of CNS pharmacology (homovanillic acid and 5-hydroxyindoleacetic acid levels in the medial septal nucleus), locomotor activity (locomotion in the open field periphery, locomotion in the open field center, locomotor activity after ethanol injection), and brain morphology (volume of the hippocampus mossy fiber pathway) all map back to the *Gsta4* locus (S1 Fig). All traits, except for homovanillic acid levels, are associated with higher expression in strains that inherited the *D* allele at this locus and are strongly and significantly correlated with *Gsta4* expression in both cortex (|*r*| > 0.52, p < 0.05) and liver (|*r*| > 0.345).

#### Multiple transcripts are modulated by variation at the *Gsta4* locus

To identify transcripts modulated by variation at the *Gsta4* locus, we queried representative expression data sets available for the BXD population for brain and peripheral tissue. In hippocampus twelve unique transcripts—*Auts2* (*A730011F23Rik*), *Zmat5* (*2610510L01Rik)*, *Csmd2* (*B230311I21*), *Dstyk* (*Ripk5*), *Nosip*, *Efs*, *Pam*, *Il17rc*, *Ino80e*, *Gm2a*, *Acap3* (*Centb5*), and *Fam60a* (*Tera*)—are regulated by the *Gsta4* locus (S2 Fig). The expression of *Efs* and *Csmd2* expression is also highly correlated (|*r*| = 0.5, *p* < 0.001) with hippocampal *Gsta4* levels. Little is known about the function of *Zmat5*, however, the remaining set of downstream genes modulated by *Gsta4* in hippocampus cover a diverse range of biological functions. Several, such as *Auts2*, *Csmd2*, *Dstyk*, *Effs*, and *Pam*, play a role in cognition and emotion based on molecular phenotypes and data from knockout mice. For example, exonic deletions in the neurodevelopmental gene *AUTS2* are often associated with cognitive deficits [50–53], and variants in *CSMD2* have been associated with comorbidity of depression and alcohol dependence [54], and schizophrenia [55]. Deletion of *Dstyk* is associated with a reduction in spatial learning and memory [56] and mice heterozygous for the cuproenzyme *Pam*—responsible for biosynthesis of ~50% of all neuropeptides—show marked deficits in thermoregulation and fear response [57]. In addition, *Effs*—a member of the CRK-associated substrate (Cas) family of adaptor proteins—is involved in assembly of large signaling complexes and may play a role in neurite outgrowth [58] and immune system function. *Gm2a* and *Il17rc* are also involved in immune signaling and response, and *Nosip* is part of the nitric oxide signaling pathway, which can be activated by oxidative stress or tissue injury. *Ino80e* and *Fam60a* are generally involved in the regulation of transcription and *Fam60* has recently been identified as a member of the Sin3 deacetylase complex involved in transcriptional repression [59].

*Cxcl2* (LOD = 2.5) is the only transcript modulated by variation in the *Gsta4* locus in the prefrontal cortex and is positively correlated with *Gsta4* expression (*r* = 0.544, *p* = .002). This gene encodes a chemokine that is induced by oxidative stress and inflammation.

In midbrain, three transcripts (*Fbxo46*, *Pigp*, *Zfp59*) are correlated with *Gsta4* expression (|*r*| > 0.4, *p* < 0.01) and map back to the *Gsta4* locus (LOD > 2.8). Respectively, these genes play a role in ubiquitination pathways, posttranslational modification of glycosylphosphatidylinositol–linked membrane proteins, and transcriptional repression.

Five transcripts map back to the *Gsta4* locus in liver (S2 Fig), including *Bet1*, *Dedd2*, *G6pc*, *Sec63*, and *Mbl1* (*Smbp)*. *Bet1*, *Dedd2*, and *Sec63* are significantly correlated with liver expression of *Gsta4* (|*r*| > 0.4, *p* < 0.05) while *Mbl1* is only modestly correlated (*r* = 0.343, *p* = 0.05) and *G6pc* is uncorrelated. With the exception of *Dedd2*, all correlations are positive. *Sec63* and *Bet1* and are involved in intracellular targeting of proteins to and from the endoplasmic reticulum (ER), respectively, and *Dedd2* is involved in apoptotic signaling and intracellular targeting of caspases [60]. *G6pc* plays a role in glucose homeostasis [61] and *Mbl1* is a soluble molecule involved in innate immunity that may also be linked to lipid metabolism through PPARα signaling pathways [62].

#### *Gsta4* and *Elovl5* are the primary candidates driving downstream trait variation

Linkage disequilibrium is a major confounding factor that limits fine-scale discrimination among physically linked candidates in a QTL from recombinant inbred populations. It is possible to achieve an eQTL precision of 1 to 4 Mb using a moderate number of BXD strains [63], however identification of the causal gene and variant responsible for downstream effects on gene expression or phenotypes is complicated by the existence of multiple cis-modulated genes residing in close proximity. Another gene, *Elovl5* (Chr 9 at 77.83 Mb), is also significantly cis-modulated (LOD > 7 in brain and LOD = 4.1 in hepatocytes) and is located within 1 Mb of the *Gsta4* locus. *Elovl5* is a metabolic gene involved in the synthesis and elongation of long chain polyunsaturated fatty acids and could also be involved in modulation of traits mapping back to this locus.

#### Correlates of *Gsta4* are enriched for metabolic pathways related to oxidative stress

Gene networks consisting of highly correlated genes can reveal underlying biological function. The top 500 correlates of *Gsta4* expression in brain (S2 Table) and liver (S3 Table) are enriched for categories related to known functions of GSTs. The results hint at shared and specialized biological functions of *Gsta4* correlates in brain and peripheral tissue, such as oxidoreductase activity in both tissues, and an emphasis on antioxidant activity and steroid metabolism in brain compared to an emphasis on glutathione and mitochondrial metabolic processes in liver.

### Gstt2

**Fig 2:**
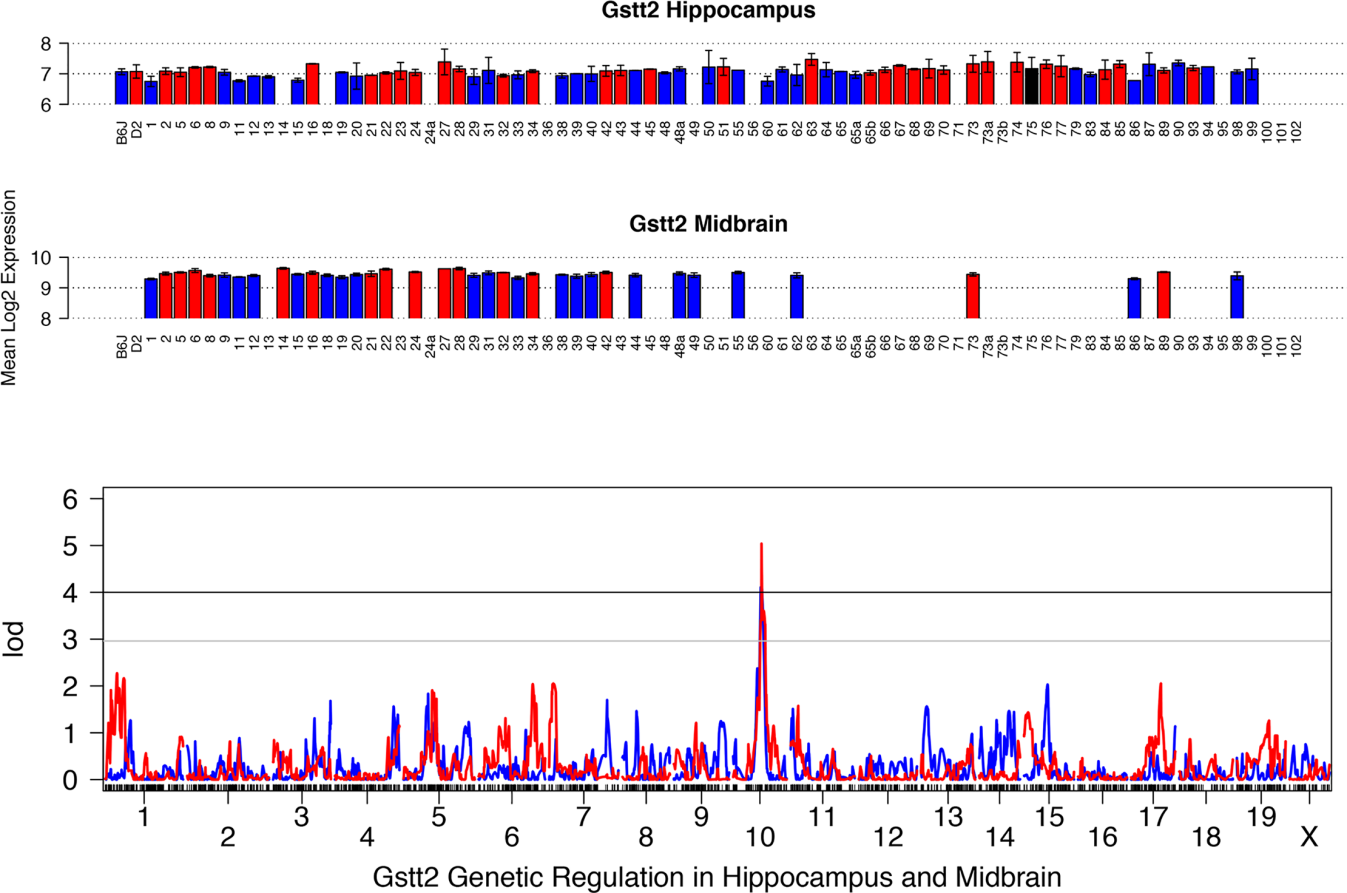
Summary of expression and QTL mapping across BXD strains for *Gstt2*. Top two panels (bar plots) show expression of *Gstt2* in each BXD strain in hippocampus and midbrain. Average log_2_ expression is shown on the y-axis and unique strains are shown on the X-axis. Red and blue indicate inheritance of the paternal *D* or maternal *B* allele of *Gsta4* in each strain, respectively. Black indicates a heterozygous (likely erroneous) genotype call. Higher expression is associated with inheritance of the *D* allele. Bottom panel shows the genetic mapping results in each tissue. Association strength (LOD) is shown on the Y-axis and plotted across the genome on the X-axis (by chromosome) for hippocampus (blue) and midbrain (red). Genome-wide significance is determined by permutation (*n* = 5000) with the threshold for significance indicated as black (significant, *p* <0.05) and grey (suggestive, *p* < 0.3) horizontal lines. Expression of *Gstt2* is modulated by variants within or near its own locus on Chr 10, a cis eQTL.

Among the BXD population expression is variable across brain regions, with average expression levels in the midbrain, and low levels of expression in the hippocampus (Fig 2). In midbrain, *Gstt2* expression variation among BXD strains is modest at nearly 1.3-fold. In hippocampus, expression is more variable at over 1.6-fold. The expression of *Gstt2* is significantly modulated by a cis eQTL in seven tissues (Table 2) from the BXD population, including the midbrain (LOD = 5.0; 1.5 LOD confidence interval on Chr 10 from 74 to 76 Mb) and hippocampus (LOD = 4.1; 1.5 LOD confidence interval on Chr 10 from 69 to 79 Mb) (Fig 2). Inheritance of the *D* allele confers higher expression in all regions. Although expression across the BXD set is higher in the liver, there is no significant cis modulation of expression detected. *Gstt2* is located in a variant poor region and, in both midbrain and hippocampus, it is the only gene within each respective 1.5 LOD confidence interval with demonstrable cis modulation of expression. Two intronic variants in *Gstt2* are segregating among the BXD population but their effect on expression is unknown. Due to the location of these variants in introns, validation by ASE analysis was not possible.

#### Multiple transcripts are downstream of the variation in *Gstt2*

No public traits in the BXD Published Phenotypes Database significantly mapped back to the *Gstt2* locus. However, hippocampal expression of *Apold1* (LOD = 2.5) and *Nfasc* (LOD = 2.4) map to the *Gstt2* locus (74.4 Mb) and both traits are negatively correlated with the expression of *Gstt2* (*r* < -0.37, *p* < 0.005) (S3 Fig). *Apold1* is an endothelial cell early response gene that may be important for endothelial cell signaling and vascular function [64] and *Nfasc* is an L1 family immunoglobulin cell adhesion molecule implicated in axon targeting and synapse formation during development [65]. In midbrain, several transcripts map back to the *Gstt2* locus at LOD > 2 and are correlated (|*r*| > 0.4, *p* < 0.01) with *Gstt2* expression (S4 Fig). These include genes with a putative role in transcription (*Aebp2*), translation (*Eif4a1*), development (*Hoxb*, *Mtpn*), cell survival (*Fgf12*), inhibition of mTOR (*Deptor*) [66], dopamine and serotonin synthesis (*Ddc*), glutamate transport (*Slc17a7*), and uptake of long chain fatty acids (*Slc27a3*).

#### Diverse phenotypes and genes enriched for metabolic processes are correlated with *Gstt2* in hippocampus

Exploration of the top 500 correlates of *Gstt2* in midbrain did not reveal any significant enrichment of biological function. However, the top correlates of *Gstt2* in hippocampus support a role in protein metabolism and cellular respiration (S4 Table). We expanded this analysis by asking whether variation in hippocampal *Gstt2* expression covaried with trait data in the BXD Published Phenotypes Database. We found a wide range of immune, morphological, and behavioral phenotypes that were significantly (*p* < 0.005) associated with variation in *Gstt2* in hippocampus, including susceptibility to Coccidiodies immitis (Valley Fever) fungal infection (record ID = 13043, *r* = 0.61, n = 30), glomerular cell counts in kidney in a long term diabetes model induced by streptozotocin treatment (record ID = 12597, *r* = -0.51, n = 35), corticosterone plasma concentration following saline injection (record ID = 10574, *r* = 0.67, n = 18), thymus weight after chronic mild stress (record ID = 13584, *r* = 0.83, n = 13), total neuron number in striatum (record ID = 13439, *r* = -0.41, n = 62), femur bone mineral density (record ID = 15967, *r* = 0.50, n = 40), and preference for 10% ethanol (record ID = 10142, *r* = 0.65, n = 19).

### Gstz1

**Fig 3:**
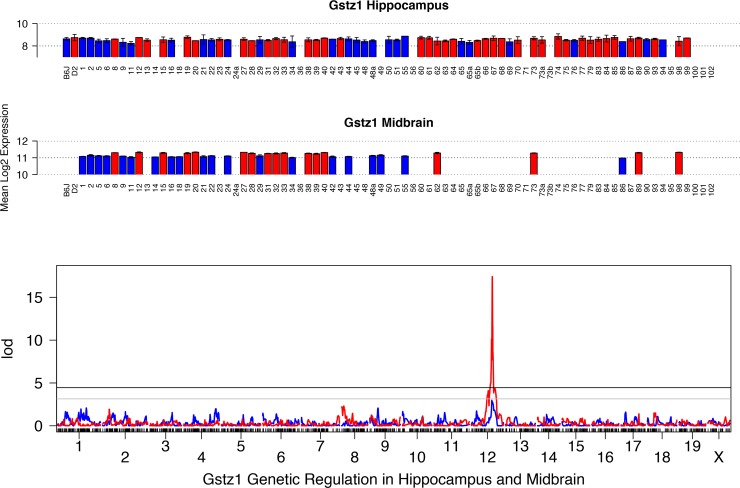
Summary of expression and QTL mapping across BXD strains for *Gstz1*. Top two panels (bar plots) show expression of *Gstz1* in each BXD strain in hippocampus and midbrain. Average log_2_ expression is shown on the y-axis and unique strains are shown on the X-axis. Red and blue indicate inheritance of the paternal *D* or maternal *B* allele of *Gstz1* in each strain, respectively. Higher expression is associated with inheritance of the ***D*** allele. Bottom panel shows the genetic mapping results in each tissue. Association strength (LOD) is shown on the Y-axis and plotted across the genome on the X-axis (by chromosome) for hippocampus (blue) and midbrain (red). Genome-wide significance is determined by permutation (*n* = 5000) with the threshold for significance indicated as black (significant, *p* <0.05) and grey (suggestive, *p* < 0.3) horizontal lines. Expression of *Gstz1* is modulated by variants within or near its own locus on Chr 12, a cis eQTL.

*E*xpression is high with a modest level of variation (1.3-fold) in midbrain while expression in the hippocampus is average and more variable with a fold change of over 1.5 (Fig 3). Cis-modulation of expression is observed in four brain tissues in the BXD set (Table 2), including midbrain (LOD = 17.4; 1.5 LOD confidence interval from 88 to 90 Mb on Chr 12) and hippocampus (LOD = 2.9) (Fig 3). In addition, a significant cis eQTL is also detected in crosses between A/J (*A*) and B6 (*B*), B6 and C3H/HeJ (*H*), and CAST/EiJ (*CT*) and B6 (Table 2). Higher expression is associated with inheritance of the *D* allele in the BXD set. In contrast, the parental allele associated with higher expression varies in other crosses, such that expression is higher for inheritance of *A* or *CT* alleles compared to the *B* allele and higher for inheritance of the *B* allele compared to the *H* allele. This pattern might be expected if several functional variants are segregating among inbred mouse strains.

*Gstz1* is polymorphic between the B6 and D2 parental strains with four SNPs in the 3’ UTR and one in an intron, however no allelic imbalance was detected for this gene in lung and liver using ASE. There was a trend towards allelic imbalance in the hippocampus with higher expression driven by the *D* allele but this was not significant (*p* = 0.15) (Table 3). *Gstz1* is located within a region that is relatively gene and variant sparse. In midbrain and hippocampus the only other cis-modulated gene is *Tmed8*, located on Chr 12 at 88.51 Mb, however, the function of *Tmed8* is not well defined.

#### Multiple transcripts are modulated by variation in *Gstz1*

No higher order phenotypes significantly map back to the *Gstz1* locus. However, in the hippocampus, expression of the pro-apoptotic gene *Scotin*, the splicing factor *Sf3b1*, and the mitochondrial inner membrane protein gene *Tmem65* are significantly modulated (LOD > 2) by variation at this locus (S5 Fig). In the midbrain, the mitochondrial gene *Cstad*, the nerve growth factor-induced early expression gene *Egr4*, the potassium large conductance calcium activated channel *Kcnma1*, and the RNA binding repressor gene *Rbm15* are also significantly modulated by variation at the *Gszt1* locus (S5 Fig).

#### Correlates of *Gstz1* suggest a role in a wide range of phenotypes and cellular metabolic processes

A diverse set of phenotypes is significantly (*P* < 0.005) correlated with *Gstz1* expression in hippocampus and midbrain. Metabolic, pharmacological, and brain electrophysiological traits, such as body temperature after a 4 g/kg injection of ethanol (record ID = 10521, *r* = -0.70, n = 16), copper level in ventral midbrain (record ID = 10729, *r* = -0.70, n = 14) and iron level in dorsal striatum (record ID = 10242, r = -0.70, n = 14), acute locomotor response to a 1.5 g/kg injection of ethanol (record ID = 10125, *r* = 0.58, n = 23), activity in the closed quadrants of the elevated zero maze after a 1.8 g/kg injection of ethanol (record ID = 12374, *r* = -0.37, n = 59), and brain activity and coherence (record ID = 17107, *r* = -0.76, n = 14), are correlated with hippocampal *Gstz1* expression. Blood chemistry and morphological traits—mean blood cell volume (record ID = 12942, *r* = 0.48, n = 35), adrenal weight (record ID = 12071, *r* = -0.45, n = 58), adrenal X-zone width (record ID = 11273, *r* = -0.37, n = 59), thalamic geniculate nuclei volume (record ID = 10934, *r* = 0.73, n = 12)—are also well correlated. Finally, traits relating to pain sensitivity, activity, and anxiety are significantly correlated with *Gstz1* expression in hippocampus, including activity after the first and second tone-shock pairing in the fear conditioning paradigm (record ID = 11914, *r* = 0.42, n = 54; 11915, *r* = 0.45, n = 54), response to mechanical nociception (record ID = 11823, *r* = -0.42, n = 55), and novel open field activity (record ID = 10916, *r* = 0.67, n = 17).

Expression of *Gstz1* in the midbrain is correlated with several pharmacological and behavioral traits including, handling induced convulsions after a 4 g/kg ethanol injection (record ID = 11382, *r* = -0.85, n = 9), acceptance of 10% ethanol solutions (record ID = 10138, *r* = -0.60, n = 20), and activity suppression to a cue in the fear conditioning paradigm (record ID = 11917, *r* = 0.50, n = 32).

The top 500 correlates of *Gstz1* expression in hippocampus and midbrain are significantly enriched for metabolic terms (S5 Table). Genes in the hippocampal *Gstz1* network were also enriched for the functional categories of rRNA binding and structural constituent of ribosome. In contrast, genes in the midbrain *Gstz1* network were enriched for transcription related terms.

### Gsto1

**Fig 4:**
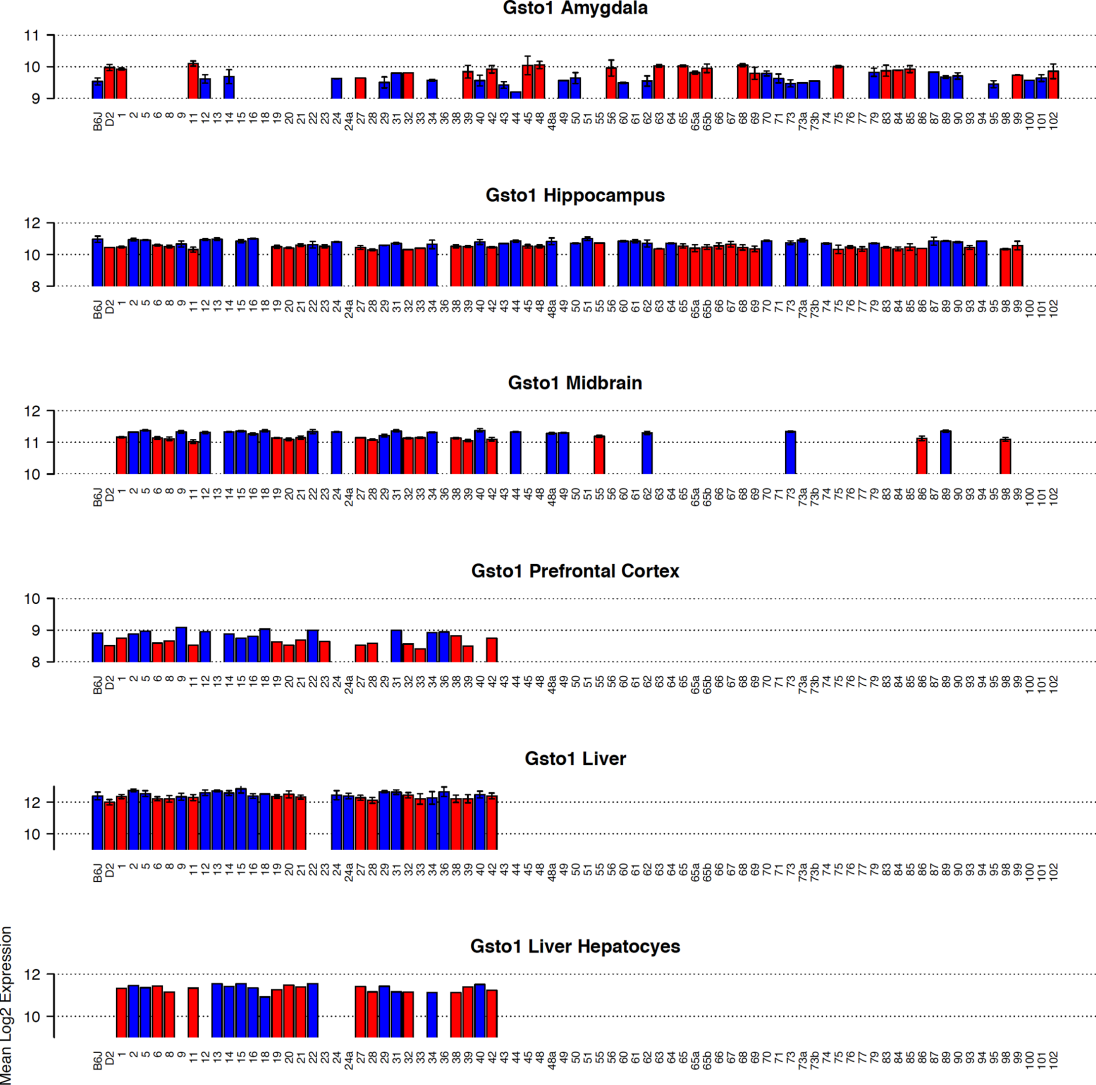
Summary of *Gsto1* expression across BXD strains. Panels (bar plots) show expression of *Gsto1* in each BXD strain in multiple tissues. Average log_2_ expression is shown on the y-axis and unique strains are shown on the X-axis. Red and blue indicate inheritance of the paternal *D* or maternal *B* allele of *Gsto1* in each strain, respectively. If only a single individual was used for expression measurements, error bars are not shown. For genetic reference populations, mapping power is derived from the number of individuals as opposed to the number of biological replicates. Higher expression is associated with inheritance of the *B* allele.

**Fig 5:**
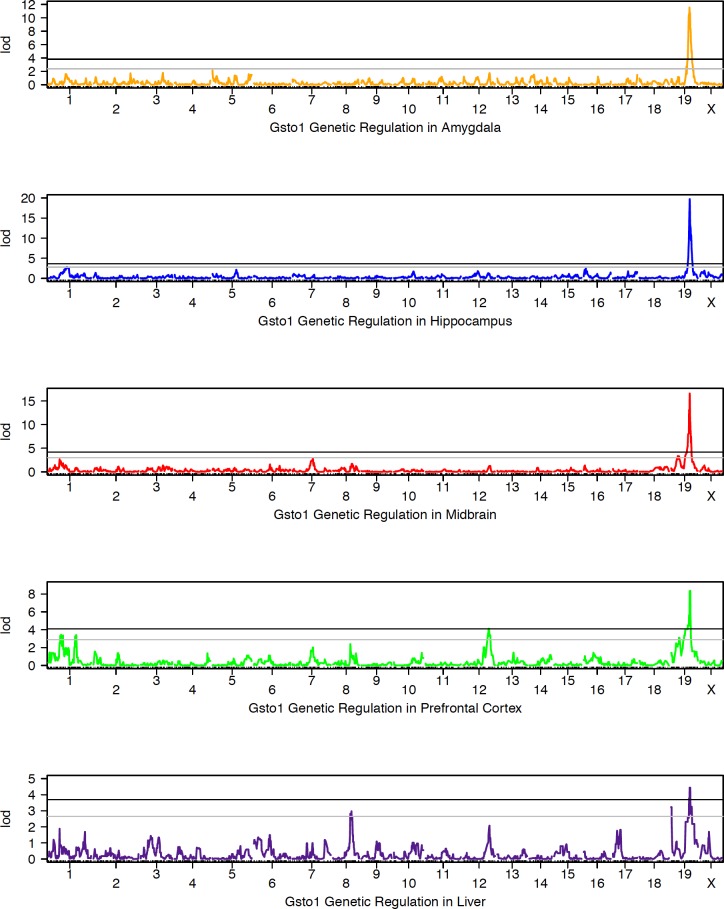
Summary of QTL mapping of *Gsto1* expression across BXD strains. Genetic mapping results are shown for brain and peripheral tissue. Association strength (LOD) is shown on the Y-axis and plotted across the genome on the X-axis (by chromosome) in each QTL map. Genome-wide significance is determined by permutation (*n* = 5000) with the threshold for significance indicated as black (significant, *p* <0.05) and grey (suggestive, *p* < 0.3) horizontal lines. Expression of *Gsto1* is modulated by variants within or near its own locus on Chr 19, a cis eQTL.

In most tissues, *Gsto1* is well expressed and variable across the BXD population (Fig 4). Nearly 60% of the variation between individual BXD strains can be explained by a genetic component (hippocampus, 1416531_at, *h*^*2*^ = 0.56) and inheritance of the *B* allele is always associated with higher expression (Fig 5). A *cis*-eQTL for *Gsto1* was consistently detected in 20 tissues from the BXD set (Table 2) The 1.5 LOD confidence interval for the *cis*-eQTL is between 47.1 and 48.0 Mb at markers rs3705264 and rs8257607 (hippocampus), precisely aligned with the position of *Gsto1*. Molecular validation of the *cis*-eQTL was performed by ASE assay in lung, liver, and hippocampus (Table 3) with the *B* allele having a significantly increased number of reads relative to the *D* allele in all tissues. Variation at the gene and mRNA level is also associated with a modest but significant 1.5–fold increase (*p* = 0.0013) in *Gsto1* protein levels in the hippocampus of B6 relative to D2 as measured by global protein quantification using tandem mass tag technology. Cis-modulation of *Gsto1* expression is also detected in crosses between A/J and C57BL/6J, Inbred Long-Sleep (ILS) and Short-Sleep (ISS), and a mouse diversity panel.

**Fig 6:**
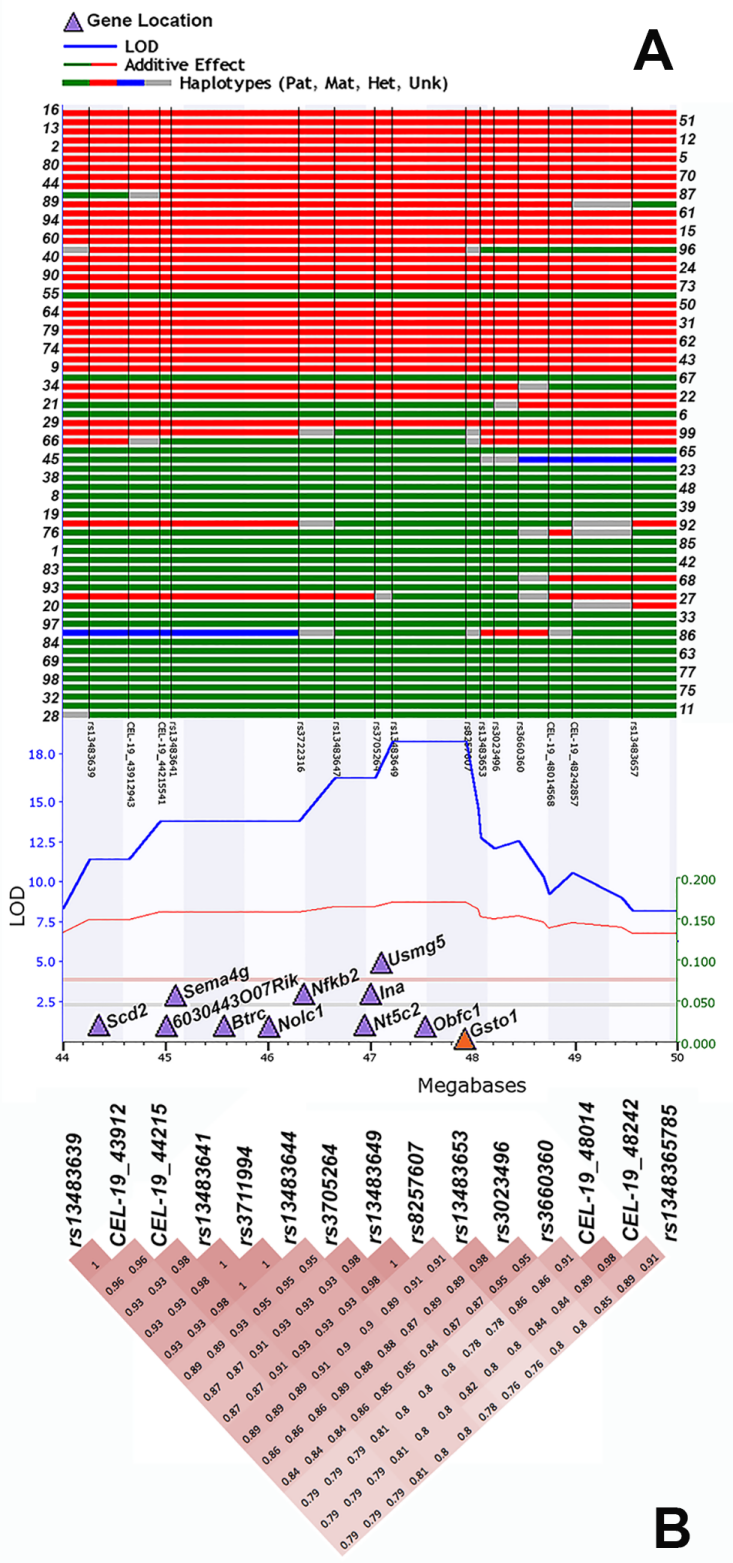
Summary of recombination and linkage disequilibrium near the *Gsto1* locus. **(A)** The association score (LOD) (y-axis) for *Gsto1* expression in the hippocampus is plotted as a solid blue line across the genome (x-axis) with horizontal lines indicating significance thresholds for significant (red) and suggestive (grey) LOD values. The physical position of *Gsto1* is indicated by the orange triangle and the locations of other cis-modulated genes are shown as light purple triangles. Haplotypes are shown above the linkage map for BXD strains with strain number shown to the right and left of the haplotypes. Vertical black lines designate marker position with marker names below the haplotype map. Red and green blocks indicate a chromosomal region inherited from the maternal B6 or paternal D2 strain, respectively. Blue areas are heterozygous and grey areas are undefined meaning that more markers would be needed to pinpoint the exact recombination breakpoint. As expected for a recombinant inbred population, this region is primarily inherited as an entire haplotype block from either parental strain. (B) Pairwise correlations between markers in the region are shown. Intensity reflects correlation strength. Markers in this region are tightly linked.

Many variants within the *Gsto1* gene are segregating among the BXD population that could account for the variation at this locus, including a 5’ UTR SNP, three SNPs located in the 3’ UTR, three synonymous variants in exons, and 47 intronic SNPs, one of which is located in a splice region. Unlike previously discussed GSTs, *Gsto1* resides within a gene- and variant-rich region that contains 10 other well expressed genes modulated by significant *cis*-eQTLs (LOD > 3.3 or LRS > 15) located within 4 Mb of the *Gsto1* locus (Fig 6). An important consideration in the analysis of *Gsto1* biological networks and downstream traits is the effect of linkage disequilibrium at this locus. For example, internexin neuronal intermediate filament protein alpha (*Ina*) is located within 1 Mb of *Gsto1* at 47.01 Mb and is associated with a strong *cis*-eQTL (1448992_at; LOD = 20). *Ina* is a major structural component of the cytoskeleton that is expressed primarily in neurons and involved in axonal architecture. Overexpression of *Ina* leads to neuronal death and degeneration in cortex, thalamus, and cerebellum [67] and is a signature of neuronal interfilament inclusion disease in humans. INA immunoreactivity is also observed in other neurodegenerative disorders, such as AD [68]. Phenotypes and genes that map into this locus, correlations between *Gsto1* and *Ina*, and correlations between other genes and *Gsto1* could result from shared biological function or result from the proximity of other *cis*-modulated genes that are in linkage disequilibrium at this locus.

#### Partial correlation analysis reveals underlying biology of *Gsto1* correlates

To address linkage near the *Gsto1* locus and identify true biological correlates of *Gsto1*, we controlled for the genetic variation at both loci (*Ina* and *Gsto1*) in hippocampus using partial correlation analysis. Residual expression correlates of *Gsto1* are likely to be valid partners and reflect underlying biology resulting from small effect loci on other chromosomes and larger network associations. We focused on the hippocampus because this tissue is especially vulnerable to neurodegeneration, for which susceptibility has been linked to polymorphisms in human *GSTO1*. We found 2797 probe sets corresponding to 2384 unique genes that are high residual correlates of *Gsto1* (p < 0.001) after partial correlation (S6 Table). In order to identify known and latent relations between co-expressed genes, we carried out a literature correlation analysis. Among these candidates, 128 unique genes shared a high co-incidence of literature citations with *Gsto1* (*r* > 0.5) (S7 Table). This gene set is highly significantly enriched (adjP < 0.001) for many metabolic GO terms as well as localization to mitochondria (S8 Table).

Multiple resources including Chillibot, Alzheimer Disease & Frontotemporal Dementia Mutation Database, the ALZGENE database, and Pubmed were used to determine whether members of the *Gsto1* coexpression network had been previously associated with AD or other neurodegenerative disorders. We identified 25 network members, including *Gsto1*, that are associated with AD in two or more databases (S6 Fig, S9 Table). Nearly half of these genes are localized to mitochondria, including *Aldh2*, *Sod2*, *Grn*, *Dlst*, *Fxn*, *Hmgcs2*, *Acat1*, *Chmp2b*, *Oat*, and *Dld*. Many genes play a role in metabolic processes, including small molecule metabolism (*Gba*, *Hmgcs2*, and *Coasy*), carboxylic acid metabolism (*Gstm1*, *Gart*, *Sod2*, *Dlst*, *Acsbg1*, *Gsto1*, and *Dld*), and oxidation-reduction processes (*Acp1*, *Blvra*, *Aldh2*, *Cygb*, and *Fxn*).

#### Correlates of *GSTO1* in human datasets support a role in mitochondrial metabolic processes

As a heterogeneous group, humans have a greater amount of genomic variation—from 10 to 40 million segregating SNPs—compared to ~5 million among the BXD family, and a much higher recombination frequency that mitigates problems with linkage disequilibrium. We leveraged a cortical gene expression data set from a mostly Caucasian population contributed by Webster and colleagues in order to explore variation and network relations. The level of *GSTO1* mRNA varies roughly 20–fold between 160 AD cases and 186 controls in cortex. There is a small (~1.2–fold) but significant (*p* = 0.0002) decrease in *GSTO1* expression in AD cases compared to controls. The top 1000 correlates of *GSTO1* in the AD data set are connected by a correlation of |0.45| or better and are also enriched for location in the mitochondrion (136 genes, adjP = 2.75e-08) as well as several other metabolic terms (S10 Table). These results support a role for *GSTO1* in mitochondrial related processes that are conserved between human and rodent and may be involved in neurodegenerative disease pathways.

#### Multiple phenotypes may be controlled by variation in *Gsto1*

Analysis of phenotypes that map back to the *Gsto1* locus in the BXD population identified morphological and pharmacological traits, including cortical grey matter volume (record ID = 10754, LOD = 2.2) and tolerance to the hypothermic effects of the dopamine D2 and D3 receptor selective agonist quinpirole (record ID = 10048, LOD = 3.7). These traits may be regulated by variation in the *Gsto1* locus or variation of neighboring cis-modulated genes.

### Mgst3

**Fig 7:**
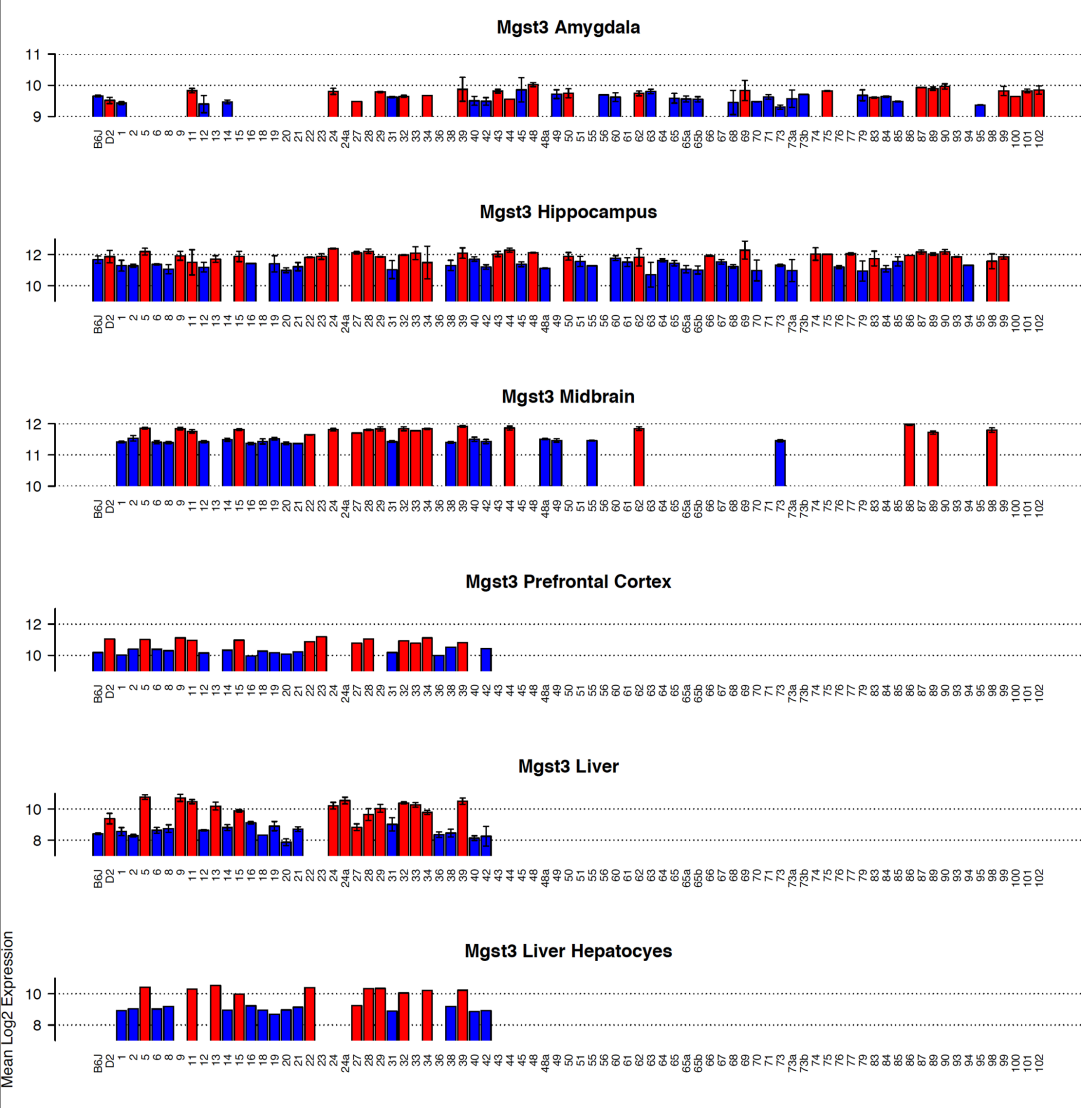
Summary of *Mgst3* expression across BXD strains. Panels (bar plots) show expression of *Mgst3* in each BXD strain in multiple tissues. Average log_2_ expression is shown on the y-axis and unique strains are shown on the X-axis. Red and blue indicate inheritance of the paternal *D* or maternal *B* allele in each strain, respectively. If only a single individual was used for expression measurements, error bars are not shown. For genetic reference populations, mapping power is derived from the number of individuals as opposed to the number of biological replicates. Higher expression is associated with inheritance of the *D* allele.

**Fig 8:**
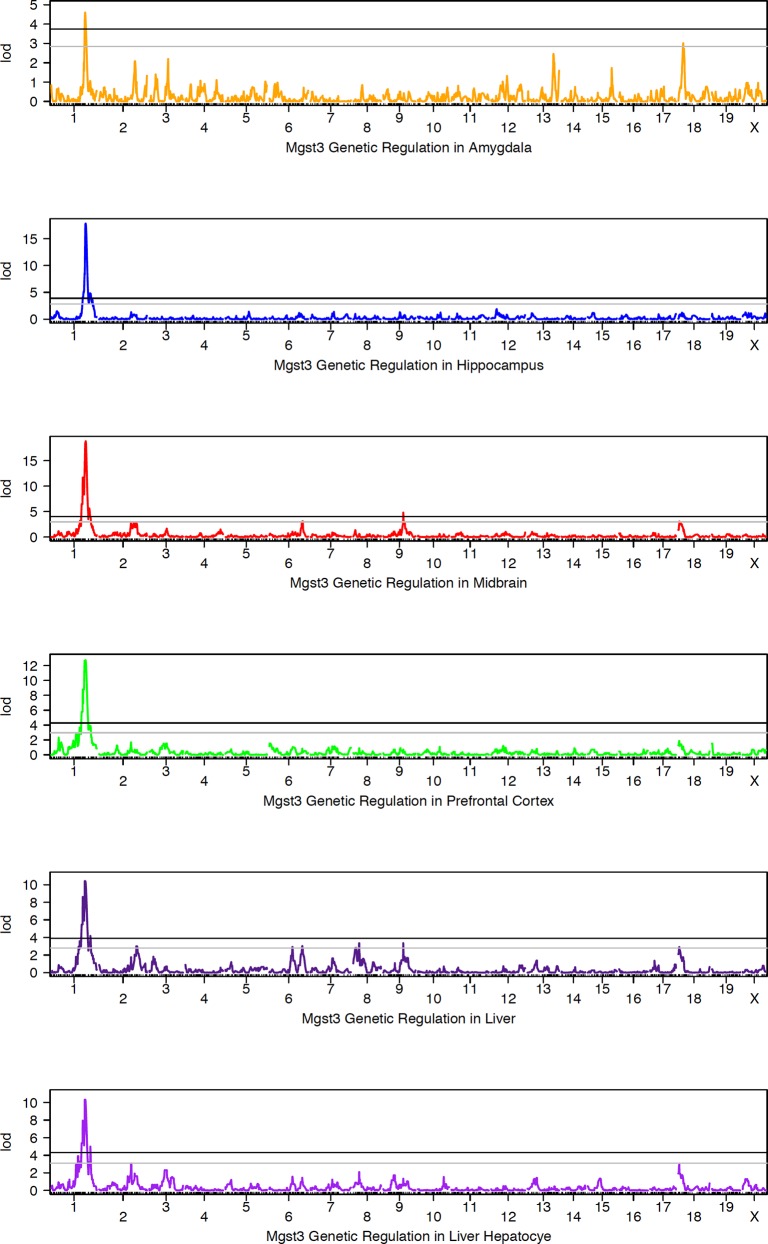
Summary of QTL mapping of *Mgst3* expression across BXD strains. Genetic mapping results are shown for brain and peripheral tissue. Association strength (LOD) is shown on the Y-axis and plotted across the genome on the X-axis (by chromosome) in each QTL map. Genome-wide significance is determined by permutation (*n* = 5000) with the threshold for significance indicated as black (significant, *p* <0.05) and grey (suggestive, *p* < 0.3) horizontal lines. Expression of *Mgst3* is modulated by variants within or near its own locus (distal Chr 1), a cis eQTL.

Expression is more robust in brain compared to liver (Fig 7). Variation across the BXD population is moderate in midbrain and amygdala (~1.5-fold), greater than 2-fold in hippocampus and prefrontal cortex, and greater than 7-fold in liver and hepatocytes (Fig 7). *Mgst3* is modulated by a strong and significant cis eQTL in 18 different tissues from the BXD set (Table 2, Fig 8). Cis modulation of expression is also detected in F2 crosses between B6 and D2 and CAST/EiJ and B6 (CTB6F2). In most data sets the 1.5 LOD confidence interval is 2 Mb and inheritance of the *D* allele confers higher expression. Inheritance of the *B* allele confers higher expression in the CTB6F2 cross.

*Mgst3* is highly polymorphic between B6 and D2 with 239 intronic and 56 UTR SNPs or small indels. Similar to the other cis-modulated GSTs, one or more non-coding variants underlie the expression variation at this locus as the cis eQTL for *Mgst3* has been validated by ASE in both liver and hippocampus (Table 3). *Mgst3* resides in a variant rich but relatively gene sparse region, although several neighboring genes (within 2 Mb of *Mgst3*) are also significantly cis-modulated (LOD > 2) in multiple data sets. This includes two genes involved in developmental processes, *Pbx1* (pre B cell leukemia homeobox 1) and *Tmco1* (transmembrane coiled-coil domains protein 1) that are cis modulated in hippocampus and midbrain and Pogo transposable element with KRAB domain (*Pogk*) that is cis-modulated in midbrain and amygdala. Immunoglobulin-like domain containing receptor 2 (*Ildr2*), an endoplasmic reticulum protein involved in lipid homeostasis, is cis-modulated in hippocampus and amygdala. No neighboring genes within 2 Mb of *Mgst3* are cis-modulated across BXD liver tissue, although transcriptional adaptor 1 (*Tada1*), which is part of the STAGA histone acetyltransferase complex, is cis-modulated in liver hepatocytes and amygdala. Although several genes are cis-modulated in one or two tissues, *Mgst3* is the only gene in this interval consistently cis-modulated across all BXD brain and liver datasets surveyed.

#### Multiple traits are modulated by variation in *Mgst3*

To investigate the biological impact of *Mgst3* variation, we identified higher order phenotypes and transcripts that mapped to within 2 Mb of the locus on Chr 1. Measures of iron, brain morphology, and activity level mapped back to the *Mgst3* locus, including iron level in spleen (record ID = 10811, LOD = 2.6), hippocampus weight (record ID = 13031, LOD = 3.9), and activity measured under a variety of paradigms and treatments (record ID = 11947, LOD = 4.3; record ID = 12370, LOD = 4.7; record ID = 12441, LOD = 3.7; record ID = 12439, LOD = 2.6; record ID = 12440, LOD = 3.9; record ID = 12409, LOD = 5.4; record ID = 12371, LOD = 4.4; record ID = 12369, LOD = 3.5).

Multiple transcripts in liver and brain are regulated from the location of *Mgst3* and are significantly co-expressed. In liver, the expression of syndecan binding protein (*Sdcbp*, *r* = 0.48), an adaptor protein thought to modulate multiple signaling pathways whose expression is altered in a number of different cancers [69], is regulated from the *Mgst3* locus. In hepatocytes, acyl-Coenzyme A dehydrogenase, medium chain (*Acadm*, *r* = 0.67), D-tyrosyl-tRNA deaylase 1 (*Dtd1*, r = 0.68), cytochrome P450, family 2, subfamily b, vertin (*Vrtn*, r = -0.66), polypeptide 23 (*Cyp2b23*, *r* = -0.71) and signal sequence receptor beta (*Ssr2*, *r* = -0.72) map back to the location of *Mgst3*. *Ssr2* is involved in targeting secretory proteins to the endoplasmic reticulum. *Acadm* is a mitochondrial flavoprotein involved in fatty acid beta-oxidation and deletion of this gene in mice is associated with fasting and cold intolerance [70]. *Cyp2b* family members are involved in metabolism and detoxification of a broad range of endogenous and exogenous substrates and *Dtd1* is involved in recycling of metabolically inactive D-amino acid tRNA molecules (e.g. D-tyrosine, D-tryptophan, D-aspartic acid) [71],. Finally, the conserved mammalian gene *Vrt* is thought to regulate fat deposition in pigs [72].

A surprising number of transcripts are partly regulated from the *Mgst3* locus in hippocampus and midbrain where 67 and 52 unique transcripts are regulated by a LOD of 2 or more, respectively. Of these transcripts, 41 are also significantly correlated (*p* < 0.01 and |*r*| > 0.3) with *Mgst3* expression in hippocampus and 52 are significantly correlated in midbrain (S11 Table). The vast majority of these correlations are negative and the only overlapping transcript is the multifaceted signaling molecule, casein kinase 1, alpha 1 (*Csnk1a1*). All downstream targets from both regions were combined into one set and the ConsensusPathDB-mouse web service (http://cpdb.molgen.mpg.de/MCPDB; [73]) was used to search for overrepresented GO, KEGG, and Reactome annotations. Few GO categories showed significant enrichment after multiple test correction; however, Signaling to p38 via RIT and RIN (*n* = 3, *q-value* = 0.04; *Raf1*, *Rit2*, *Hras1*), RAF activation (*n* = 2, *q-value* = 0.04; *Raf1*, *Hras1*), and L-ascorbate biosynthesis VI (*n* = 2, *q-value* = 0.04; *Ugp2*, *Akr1a1*) were significantly enriched in the list of downstream targets. In contrast, no significantly modulated downstream transcripts were detected for prefrontal cortex and, of the five transcripts detected in amygdala (*Afg3l1*, *Cplx1*, *Peli3*, *Rpl30*, *Sh3bp5l*), only mitochondrial targeted ATPase family gene 3-like 1 (*Afg3l1*) and complexin 1 (*Cplx1*), a modulator of synaptic vesicle release, is positively correlated with *Mgst3*.

#### Correlates of *Mgst3* in mouse and human populations suggest a role in energy production

Similar to *Gsto1*, linkage disequilibrium confounds attempts to definitively assign subsets of downstream genes and phenotypes to variation in *Mgst3*. As linkage disequilibrium is reduced in human populations we compared the top 500 correlates of *Mgst3* in mouse and human brain and found them to be enriched for GO categories associated with energy production and metabolism. In BXD midbrain, the top correlates are enriched for ATPase activity coupled to transmembrane movement of substances and mitochondrial proton-transporting ATP synthase complex (S12 Table). The top correlates in 187 normal aged human cortical samples are enriched for respiratory electron transport chain, ATP biosynthetic process, mitochondrion, and mitochondrial inner membrane (S13 Table).

### GST superfamily expression is correlated in both brain and peripheral tissue

**Fig 9:**
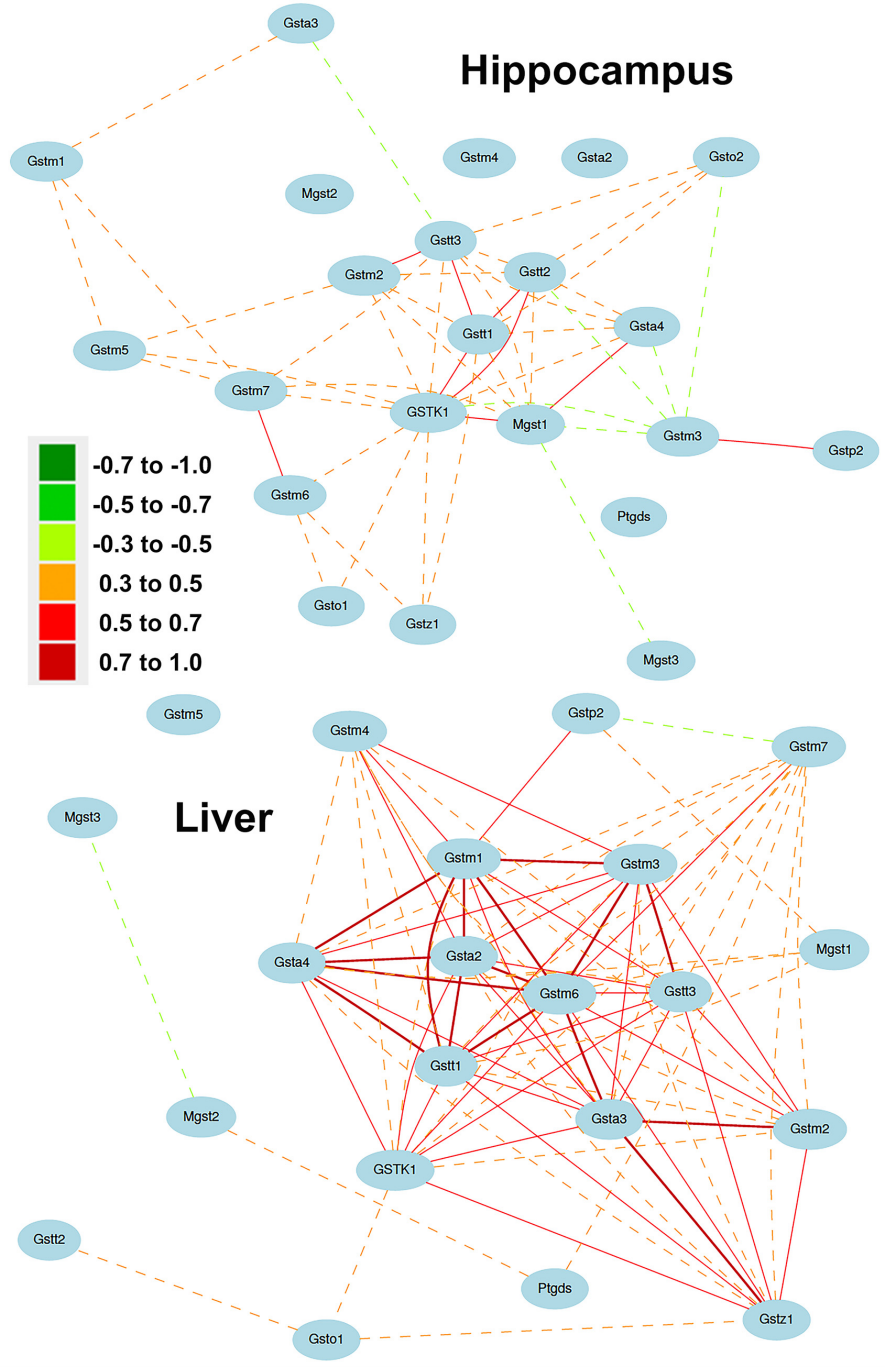
GST coexpression networks in brain and liver. Top and bottom panels show GST coexpression networks in hippocampus and liver, respectively. Positive correlations are indicated by warm line (edge) colors and negative correlations are indicated by cool edge colors. In addition, correlations greater than |0.7| are indicated as bold lines and those less than |0.5| are indicated by dashed lines. All correlations are greater than |0.3|. The expression of many GST genes is positively correlated.

In addition to exploring the genetic regulation of individual GSTs, we also explored interactions among all family members and found that there is extensive covariation in hippocampus and liver. Covariation can be driven by shared biological function, transcriptional regulation, or genetic regulation. Close physical proximity on chromosomes can drive coexpression through common transcriptional control. Paralogs of the alpha, mu, omega, and theta class are located in gene clusters on Chrs 9 (78 Mb), 3 (107.7Mb), 19 (47.9 Mb), and 10 (75.2 Mb), respectively. Theta class members *Gstt1*-*3* demonstrate robust expression covariation and Mu class members *Gstm1*, *Gstm5*, *Gstm7*, and *Gstm2* are also well correlated (Fig 9). In contrast, Alpha and Omega paralogs do not display extensive covariation. No common genetic mechanism—such as a single regulatory locus or quantitative trait loci (QTL)—was detected that modulates the expression of multiple GSTs, suggesting that coexpression in these tissues is primarily driven by shared biological function and close physical proximity.

In hippocampus, nearly all members of the GST network are connected by at least two edges (|*r*| > 0.3) with the exception of *Gsta2* and genes with low expression in hippocampus (*Ptgds*, *Mgst2*, and *Gstm4*; Fig 9). Correlations in the hippocampal GST network are mostly positive with the notable exception of most edges connecting *Gstm3*. Several GSTs, including *Gstk1*, *Gstt1-3*, and *Mgst1*, are network hubs with connectivity greater than seven nodes. The expression of *Gstm2*, *Gstt1*, *Gstk1*, *Mgst1*, and *Gsta4* is tightly correlated and these genes are highly interconnected. The first principle component (PC1) of this module explains nearly 60% of the covariation in expression and was used as a signature to find additional highly correlated genes in hippocampus. The top 500 genes in this GST network are significantly enriched for localization to the mitochondrion and many metabolic and antioxidant related process (S14 and S15 Tables). Taken together these results suggests that clustering of physically unlinked GST family members in brain is most likely due to underlying biology and the role of these genes in energy production and metabolic homeostasis.

Expression covariation of GSTs is even more pronounced in liver (Fig 9). Network connections are overwhelmingly positive and there is a much higher degree of network connectivity when compared to brain. *Gstm5* is unconnected from the network at a threshold of |*r*| > 0.3 and genes with low expression—*Mgst3* and *Ptgds*—are not well connected. Interestingly, positive and negative correlations are not well conserved between the hippocampus and liver network and may result from differences in expression level, which tends to be higher in liver, or tissue heterogeneity, which is greater in brain. However, comparison of tightly correlated but physically unlinked GSTs in liver revealed the same underlying network biology. *Gstm1*, *Gsta2*, *Gstm6*, *Gstt1*, *Gstk1*, and *Gstz1* cluster tightly and PC1 captures over 70% of the variance in coexpression. The top 500 correlates of the GST network signature in liver are also significantly enriched for localization to the mitochondrion and metabolic terms (S16 and S17 Tables). Despite the strong overlap of biological function associated with both GST networks there is only about a 9% overlap between the top correlates in liver in brain, suggesting tissue specific expression of correlated metabolic genes in these tissues.

## Discussion

*T*he GST family of enzymes utilize glutathione and contribute to the biotransformation and disposition of many compounds, including drugs, pesticides, carcinogens, and products of oxidative stress. Alterations in the function of these genes may act as a risk factor for disease. Here, we have investigated the genetic regulation of GSTs and addressed the potential impact of naturally occurring variation in one component of the glutathione system. Our study is the first such attempt to systematically investigate the cause and consequences of variation in GSTs using a diverse genetic population.

Similar to human populations, the BXD family exhibits a wide range of variation in the expression of GST family members. As expected, physically linked paralogs, such as members of the Theta and Mu class, demonstrate coordinately regulated expression. However, many unlinked GST family members are coexpressed in brain and peripheral tissue and the top correlates of each network are significantly enriched for metabolic terms, confirming that GSTs have broad and overlapping roles in cellular metabolic processes. The covariation between members of the GST network is primarily driven by this underlying biological function as no strong QTLs associated with network coexpression were detected under baseline conditions. In some ways this result is surprising since many genes involved in oxidative stress pathways, including GSTs, are known to contain antioxidant response elements in their promoters [2] and are regulated by the *Nrf2* antioxidant response element signaling pathway under both basal and stress conditions [74]. *Nrf2*, commonly known as *Nfe2l2*, is actually well correlated (*r* = 0.6, *n* = 67, *p* = 3.77E-08) with expression of the GST network in hippocampus (S2 Table) but does not show cis-modulation of expression in the BXD population. In the absence of strong genetic variants in key control genes, *Nfe2l2* and other transcriptional regulators likely work together to modulate baseline expression of GSTs and other metabolic and antioxidant response genes and it is this shared biology that drives network correlations. Although beyond the scope of this paper, a detailed analysis of transcription factors correlated with GST networks across tissues may reveal additional general or tissue-specific transcriptional regulators of genes involved in the response to antioxidants or metabolic stress.

Based on careful genetic analysis of individual GST expression across the BXD population, we identified five GSTs, *Gsta4*, *Gstt2*, *Gstz1*, *Mgst3*, and *Gsto1*, whose expression is regulated by distinct and local sequence variants in one or more tissues. We were able to replicate and validate cis eQTLs for *Gsta4*, *Gsto1*, and *Mgst3* using RNA-seq based ASE assays. This finding is significant because mutations in these genes have a functional impact on transcript expression and have the potential to modulate additional traits. The wealth of multiscalar trait data accumulated since the late 1970’s for the BXD family allowed for a detailed interrogation of the effect of segregating mutations in all five genes.

### Minimal obvious impact of *Gstt2* and *Gstz1* variants

The cis eQTLs in *Gstt2* and *Gstz1* could not be confirmed by ASE due to the lack of an informative coding variant and relatively low hippocampal expression, respectively. Both GSTs reside in relatively gene or variant sparse regions and are the only genes within each interval with local modulation of expression. Coding mutations—missense, stop, or deletions—could alter protein function without a concomitant change in gene expression and, consequently, would not be identified as cis eQTLs. A search for high impact coding variants in all genes located within each respective QTL confidence interval was conducted for *Gstt2* and *Gstz1* using inbred strain genomic sequence data available and annotated by the Wellcome Trust Sanger Institute (http://www.sanger.ac.uk/sanger/Mouse_SnpViewer/rel-1303; [45]). This analysis did not reveal any genes within the confidence interval with potentially damaging coding variants, except for a frameshift mutation in a predicted gene model *Gm6803* located in the *Gstz1* interval. *Gstt2* and *Gstz1* are therefore good candidates for modulating any traits that map back to the vicinity of each gene. However, these genes appear to exert only a subtle effect on phenotype with few transcripts or higher order traits mapping back to either locus. Variation in *Gstt2* or *Gstz1* may be buffered by the activities of other GSTs and genes with overlapping functions, or an effect may only be evident after a challenge to the system.

Compensation or buffering is an important consideration for *Gstt2* as there are multiple highly related Theta class members in the mouse genome. *Gstt1* and *Gstt2*, for example, share greater than 50% sequence homology in both rodents and humans. However, in humans there are only two theta class members, compared to four in mice, and the locus is very complex; GSTT1 is frequently deleted [75–78] and an inverted duplication of GSTT2 leads to the transcription of pseudogenes whose function is not well defined [76,79]. While several studies have reported associations between GSTT2 and differential cancer susceptibility [79–81], the potential role of this gene is still unclear due to the structural complexity of this locus in humans. Several targeted mutations of murine *Gstt2* are available, however, phenotypic abnormalities have yet to be reported (Mouse Genome Informatics, www.informatics.jax.org). The lack of a strong phenotype upon deletion is consistent with our lack of evidence for direct genetic control over more complex traits, however, multiple transcripts are modulated from the *Gstt2* locus in the BXD population. These genes play a role in development and cell growth including, *Apold1*, *Aebp2*, *Eif4a1*, *Hoxb*, *Mtpn*, *Fgf12*, *Deptor*, *Nfasc*. Our genetic dissection of transcripts modulated by variation in *Gstt2* in the BXD population suggests a plausible link between regulation of cell growth pathways and cancer GWAS [79–81] and further investigation is warranted.

The failure to associate robust trait variation to segregating mutations in *Gstz1* is perhaps especially surprising as this gene has no paralogs and plays a distinct role in both rodents and humans in the catabolism of phenylalanine and tyrosine [82] and in the biotransformation of dichloroacetate (DCA, a chemotherapeutic agent that also inactivates *Gstz1*) to glyoxylate [83]. Indeed, deletion of this gene in mice leads to upregulation of alpha, mu, and pi class GSTs and is also associated with increased sensitivity to diets high in phenylalanine and tyrosine [84,85]. These strong effects are puzzling given our findings, however, naturally occurring variants in regulatory regions are expected to result in smaller expression changes than complete gene deletion and may therefore exert a more modest influence on phenotype. In humans there are common haplotypes in *GSTZ1* that could affect gene function [86], although the implications on metabolic function or pharmacokinetics of drug metabolism remain unclear [11]. The BXD family would thus be ideally suited for targeted dietary or toxicological studies to evaluate potential consequences of naturally occurring variation in *Gstz1*.

### Strong and consistent variation in *Gsto1* levels is not associated with major phenotypic abnormalities

Both members of the Omega class are conserved between human and rodent but only *Gsto1* demonstrates significant cis-modulation of expression in the BXD population. Interestingly, this variation has only modest effects on downstream trait variation. In humans, a locus on human chromosome 10q that contains both *GSTO1* and *GSTO2* has been implicated in risk and age-at-onset of AD [87–89]. Li and colleagues also detected significantly lower *GSTO1* levels in the hippocampus of AD patients compared to controls, and alleles of *GSTO1* are significantly associated with age-at-onset for AD and Parkinson’s disease (PD) [88]. Despite these findings, several follow-up studies have been published with mixed results [90–93]. GSTO1 is also thought to play a role in inflammation [94], arsenic and alpha-haloketone biotransformation [95,96], and reduction of S-(phenacyl)glutathiones, such as S-(4-nitrophenacyl)glutathione [96]. However, deletion of *Gsto1* in mice does not produce an observable phenotype [97] and GSTO1 deficiency in a human breast cancer cell line does not alter sensitivity to arsenic or cytotoxic cancer drugs [95] Despite these discordant findings *Gsto1* expression is highly correlated with many metabolic genes, some of which are involved in AD pathways. Among the 2384 unique gene correlates of *Gsto1* expression in hippocampus are 128 genes that share a high co-incidence of literature citations—24 of which are known to be associated with AD or dementia, including well studied candidates *Chmp2b*, *Optn*, *Dlst*, *Sod2*, and *Acat1 [98–107]*. *GSTO1* expression in the cortex of human AD and control cases is highly variable and a significant decrease in *GSTO1* levels is evident among AD cases. In both human (AD and control cases) and mouse *GSTO1* correlation networks we found significant enrichment for mitochondrial genes. Although probably not the main genetic driver, *GSTO1* could interact with other variants, environmental exposure, and aging processes to influence AD risk and pathology, perhaps through changes in glutathione regulation, metabolic processes, or mitochondrial function. Mitochondrial dysfunction has long been implicated in a neurodegenerative disorders such as AD, PD, Huntington’s disease, and amyotrophic lateral sclerosis [108,109]. A direct link between *GSTO1* and mitochondrial function has not yet been reported but is suggested in both our human and mouse brain expression networks and further investigation is warranted

### Variants in *Gsta4* exert a downstream effect on trait variation

We found that expression of *Gsta4* is variable and modulated by local sequence variants in the BXD and other murine populations. In primates and rodents, *Gsta4* has a high affinity for a major byproduct of lipid peroxidation, 4-hydroxynon-2-enal (4-HNE) [110]. Toxic by-products of lipid peroxidation such as 4-HNE act as signaling molecules and markers of peroxidation that are often elevated after tissue injury or in inflammatory and degenerative diseases, including Parkinson’s Disease [111]. There is also evidence that higher levels of *Gsta4* may protect against neurodegeneration, especially after traumatic brain injury, which is associated with elevated levels of 4-HNE [112]. Non-coding variants segregate among human populations, but it remains to be seen how these impact gene expression [113]. However, variation at GSTA4 has been associated with epilepsy remission following pharmacological intervention [114], suggesting a possible role in drug metabolism in addition to a putative neuroprotective role. *Gsta4* null mice show a range of tissue-specific phenotypes, including susceptibility to oxidative stress [110], CCl4 hepatotoxicity [115], and increased mitochondrial dysfunction and protein carbonylation in adipose tissue [116].

Based on the biological function of *Gsta4* and the strong phenotype after deletion, we expected *Gsta4* variation to exert a downstream effect on gene expression and higher order phenotypes in the BXD panel. Indeed, our results support both known and novel functions. For example, *Cxcl2* (prefrontal cortex), *Bet1* (liver), and *Nosip* (hippocampus) are modulated by variation in *Gsta4* and are involved in response to lipid peroxidation. In *Gsta4* knockout mice, exposure to an alcohol liquid diet results in increased lipid peroxidation and expression of multiple inflammatory markers, including *Cxcl2 [117]*. *Bet1* expression has been shown to increase during ER stress [118], which can be induced by various insults, including accumulation of misfolded proteins and by lipid oxidation byproducts such as 4HNE [119]. Combination of superoxide and the signaling molecule nitric oxide (NO) can generate peroxynitrite anions that damage membranes and induce lipid peroxidation [120]. Of note, *Nosip* is a negative regulator of the main enzyme (nitric oxide synthase) responsible for production of NO [121].

Other downstream targets of *Gsta4* hint at an overlapping but undefined role in response to oxidative stress and include genes involved in apoptosis (Dedd2), ubiquitination (*Fbxo46*), transport into the ER (*Sec63*) transcriptional regulation (*Zfp59*, *Ino8e*, *Fam60a*), immune function (*Gm2a*, *Effs*, *Ill7rc*) and protein targeting to membranes (*Pigp*). In addition to a role in immune function, *Gm2a* is also thought to bind and traffic a range of lipid targets, including phosphatidylcholine [122] and could be involved in repair pathways in response to lipid peroxidation.

Finally, several of the gene products that mapped back to the *Gsta4* locus in hippocampus are thought to be involved in cognition and behavior (*Aust2*, *Csmd2*, *Dstyk*, *Pam*). Interestingly, a number of traits related to hippocampal activity and behavior were also partially modulated by variation in *Gsta4*, such as hippocampal mossy fiber pathway volume, dopaminergic activity (homovanillic acid levels) in the medial septal nucleus, and several measures of locomotor activity. The medial septal nucleus is innervated by dopaminergic projections from the ventral tegmental area [123] and projects to the hippocampal formation. Stimulation of this ascending pathway produces theta wave activity in the hippocampus of rodents [124] that is critical for information processing and memory storage during exploratory behavior [125,126]. These downstream traits suggest a novel role of *Gsta4* in exploratory activity, perhaps mediated through the septal-hippocampal pathway.

Although these findings are exciting and warrant further investigation, a caveat of this analysis is linkage disequilibrium and the size of the haplotype blocks in the BXD population. A neighboring gene involved in fatty acid synthesis, *Elovl5*, is also modulated by a cis eQTL and could be responsible for variation in traits mapping to this locus on chromosome 9. *Elovl5* deletion results in female infertility, increased levels of liver triglycerides, and hepatic steatosis [127] with no effect on body weight or other phenotypic abnormalities. In contrast *Gsta4* knockout mice exhibit a range of phenotypic alterations including, reduction of 4-HNE conjugating activity and higher baseline levels of 4-HNE [110,115], lower litter size, increased incidence of bacterial infections, greater susceptibility to the herbicide paraquat, higher fat content in bone marrow [110], mitochondrial defects [116] and extended lifespan [128]. The higher burden of phenotypic abnormalities in the *Gsta4* knockout mouse and the overlapping roles in lipid peroxidation pathways of downstream targets identified in our study strongly suggest that the majority of traits mapping back to the chromosome 9 locus are indeed modulated primarily by variation in *Gsta4* and not *Elovl5*.

### Variants in *Mgst3* exert a downstream effect on trait variation

The only membrane-associated microsomal GST family member with consistent genetic modulation of expression among the BXD population is *Mgst3*. Although not as well studied as some of the other cis-modulated genes, variation has previously been linked to hippocampal size in the BXD population [129]. In addition, activation of stress response transcriptional activators such as PPARα or *Nrf2* lead to increased expression of *Mgst3* [130] and studies have documented increased *Mgst3* expression in liver after caloric restriction [131] and in heart in response to opioidergic preconditioning that is protective against ischemia-induced injury [132]. Conversely, downregulation of *Mgst3* has been observed in Alzheimer’s disease [133]. These studies suggest that *Mgst3* has overlapping roles with other GSTs in oxidative stress pathways, unlike many of the other MAPEG family members primarily involved in inflammatory pathways [2].

Downstream trait analysis in the BXD family supports involvement of *Mgst3* in detoxification and oxidative stress pathways and hints at novel roles in pathways involved in cancer, cell growth, and behavior. In addition to hippocampal size, multiple measures of locomotor activity are modulated from the *Mgst3* locus, suggesting that this gene may also play a role in circuitry or metabolism of brain regions involved in exploratory behavior. In liver, nearly all transcripts whose expression is modulated by *Mgst3* are involved in some aspect of metabolism or detoxification (*Acadm*, *Cyp2b*, *Dtd1*, *Vrt*), with the notable exception of *Sdcbp* which is a signaling molecule implicated in tumor growth and metastasis for melanoma [134], hepatoma [135], lung cancer [136], glioma [137], urothelial cell carcinoma [138], and breast cancer [139]. Hundreds of transcripts map back to the *Mgst3* locus in brain, with 67 and 52 modulated in hippocampus and midbrain, respectively. Among these, only *Csnk1a1* was significantly modulated by variation in *Mgst3* in both regions. *Csnk1a1* is known to regulate the *Wnt* signaling pathway [140], which is frequently disrupted in cancer and modulates cell growth, adhesion, and survival. Loss of function of *Csnk1a1* may be related to poor prognosis in colon cancer [141,142]. In addition, alterations in *Csnk1a1* function or expression have been implicated in prostate cancer [143], leukemia [144], myelodysplatic syndrome [145], and breast cancer [146]. The large set of genes that are partially controlled by variation in *Mgst3* in hippocampus and midbrain are significantly enriched for signaling to p38 via RIT and RIN, RAF activation and L-ascorbate biosynthesis. The P38 signaling pathway is activated by environmental stress, oxidative stress, and cytokine signaling [147,148]. Activation of the Ras/Raf/MEK/ERK signaling pathway is associated with cell growth and survival, and is often altered in oncogenesis [149]. Taken together, systems genetics analysis across the BXD family supports a role for *Mgst3* in oxidative stress and metabolic pathways and suggests a possible novel role in cell growth signaling pathways often disrupted in many cancers.

### Conclusion

GSTs are part of a larger cell surveillance system involving GSH. This system depends on the actions of genes involved in GSH synthesis (e.g. glutamate cysteine ligase and glutathione synthetase), export of GSH conjugates from the cell by multidrug resistance-associated proteins, and a whole host of enzymes involved in scavenging reactive oxygen species (ROS) and removal of toxic byproducts of cellular respiration, such as aldehyde and alcohol dehydrogenase, catalase, glutathione peroxidase, and superoxide dismutase [2]. Understanding the genetic architecture of GSTs and other members of the GSH surveillance system in the BXD family is critically important for modeling human disease and pharmacokinetics in murine populations and for evaluating the biological impact of these genes. Multiple GSTs, including *Gsta4*, *Mgst3*, *Gstt2*, *Gstz1*, and *Gsto1* are variable among the BXD population with some individuals carrying mutations that alter gene expression. Future studies can leverage this variation in the BXD cohort to test the influence of these variants in response to perturbations such as exposure to drugs or toxins, cancer, or neurodegeneration, or to evaluate novel associations in human GWAS. Here we used an integrative translational approach to characterize gene regulation and co-expression, and to identify potential downstream targets of GST genes. Our analysis provides new insight into the biological function of GSTs and possible mechanistic links to disease.

## Supporting Information

S1 Fig Phenotypes mapping back to the *Gsta4* locus. Genetic mapping results are shown for traits from the BXD Published Phenotypes Database. Association strength (LOD) is shown on the Y-axis and plotted over a 30 Mb region spanning the *Gsta4* cis eQTL. Line colors show results of interval mapping for each trait plotted on the same scale according to the legend (upper left corner). Homovanillic acid levels (record ID = 12801, LOD = 2.7) and 5-hydroxyindoleacetic acid levels (record ID = 12800, LOD = 2) in the medial septal nucleus, locomotion in the open field periphery (record ID = 11862, LOD = 4.7), locomotion in the open field center (record ID = 11868, LOD = 3.8), locomotor activity after ethanol injection (record ID = 11703, LOD = 4.7), and volume of the hippocampus mossy fiber pathway (record ID = 12591, LOD = 2.1).
(PNG)

S2 Fig Transcripts mapping back to the *Gsta4* locus. Genetic mapping results are shown for nine of the twelve transcripts regulated by the *Gsta4* locus in hippocampus (top panel) and for 5 transcripts in liver (bottom panel). *Auts2 (A730011F23Rik)*, *Zmat5 (2610510L01Rik)*, and *Csmd2 (B230311I21)* are not shown. Mapping of *Gsta4* is shown in dark blue in the bottom panel in liver for reference. Association strength (LOD) is shown on the Y-axis and plotted over the region spanning the *Gsta4* cis eQTL. Line colors show results of interval mapping for each trait plotted on the same scale according to the legend (upper left corner) in each panel.
(TIF)

S3 Fig Transcripts mapping back to the *Gstt2* locus in hippocampus. Top panel shows correlations between *Gstt2* and transcripts mapping back to the *Gstt2* locus. Bottom panel shows genetic mapping results for each transcript and *Gstt2* (for reference, in red) in hippocampus. Association strength (LOD) is shown on the Y-axis and plotted over the region spanning the *Gstt2* cis eQTL. Line colors show results of interval mapping for each trait plotted on the same scale according to the legend (upper left corner) in each panel.
(TIF)

S4 Fig Transcripts mapping back to the *Gstt2* locus in midbrain. Top panel shows correlation network between *Gstt2* and transcripts mapping back to the *Gstt2* locus. Positive correlations are indicated by warm line (edge) colors and negative correlations are indicated by cool edge colors. Network threshold is r = |0.3|. Bottom panel shows genetic mapping results for each transcript and *Gstt2* (for reference, in red) in midbrain. Association strength (LOD) is shown on the Y-axis and plotted over the region spanning the *Gstt2* cis eQTL. Line colors show results of interval mapping for each trait plotted on the same scale according to the legend (upper left corner) in each panel.
(TIF)

S5 Fig Transcripts mapping back to the *Gstz1* locus. Genetic mapping results are shown for each transcript in hippocampus and midbrain. Association strength (LOD) is shown on the Y-axis and plotted over the region spanning the *Gstz1* cis eQTL. Line colors show results of interval mapping for each trait plotted on the same scale according to the legend (upper left corner).
(TIF)

S6 Fig *Gsto1* AD coexpression network. Network for the 25 AD associated genes that are correlated with hippocampal *Gsto1* expression and co-cited in the literature. Positive correlations are indicated by warm line (edge) colors and negative correlations are indicated by cool edge colors. Network threshold is set at r = |0.3|. The center of the network is the *Gsto1* residual trait generated after partial correlation analysis. All 25 genes are highly connected in this network.
(TIF)

S1 Table Probe set summary. (XLSX)

S2 Table Significantly enriched GO Categories in the top 500 correlates of *Gsta4* in hippocampus. (XLSX)

S3 Table Significantly enriched GO Categories in the top 500 correlates of *Gsta4* in liver. (XLSX)

S4 Table Significantly enriched GO Categories in the top 500 correlates of *Gstt2* in hippocampus. (XLSX)

S5 Table Significantly enriched GO Categories in the top 500 correlates of *Gstz1* in the hippocampus and midbrain. (XLSX)

S6 Table Partial correlates of *Gsto1* in the hippocampus. All have partial r (p-value) < 0.001. Below background expression is shaded.
(XLSX)

S7 Table Partial correlates of *Gsto1* in the hippocampus and literature co-citations. All have partial r (p-value) < 0.001 and literature (lit) r > 0.5. Below background expression is shaded.
(XLSX)

S8 Table Gene enrichment for top 128 genes correlated with *Gsto1* expression in hippocampus and literature co-citations. (XLSX)

S9 Table Co-incidence of literature citations among top 128 genes and AD. Shading denotes genes whose expression in the hippocampus is below background (average log2 expression < 7). Lit Corr = literature correlation between the genes listed in the table and *Gsto1* based on the NCBI Rif; AD & FTDM DB = Alzheimer and Frontotemporal Dementia Mutation Database; Alzgene = field synopsis of genetic association studies in AD.
(XLSX)

S10 Table Gene enrichment for mitochondrion cellular component for the top 1000 gene correlates of GSTO1 using cortical expression data from human AD cases and controls. (XLSX)

S11 Table Downstream targets of *Mgst3*. (XLSX)

S12 Table Gene enrichment for top 100 correlates of *Mgst3* in mouse. (XLSX)

S13 Table Gene enrichment for top 500 correlates of *Mgst3* in human. (XLSX)

S14 Table Top 500 correlates of hippocampal GST network PC1. (XLSX)

S15 Table Gene enrichment for top 500 genes correlated with hippocampal GST network PC1. (XLSX)

S16 Table Top 500 correlates of liver GST network PC1. (XLSX)

S17 Table Gene enrichment for top 500 genes correlated with liver GST network PC1. (XLSX)
