# Supplementary figures and images for: The Genetic Architecture of Murine Glutathione Transferases
Source: PLoS One. 2016 Feb 1;11(2):e0148230. doi: 10.1371/journal.pone.0148230 (PMC4734686; doi:10.1371/journal.pone.0148230)

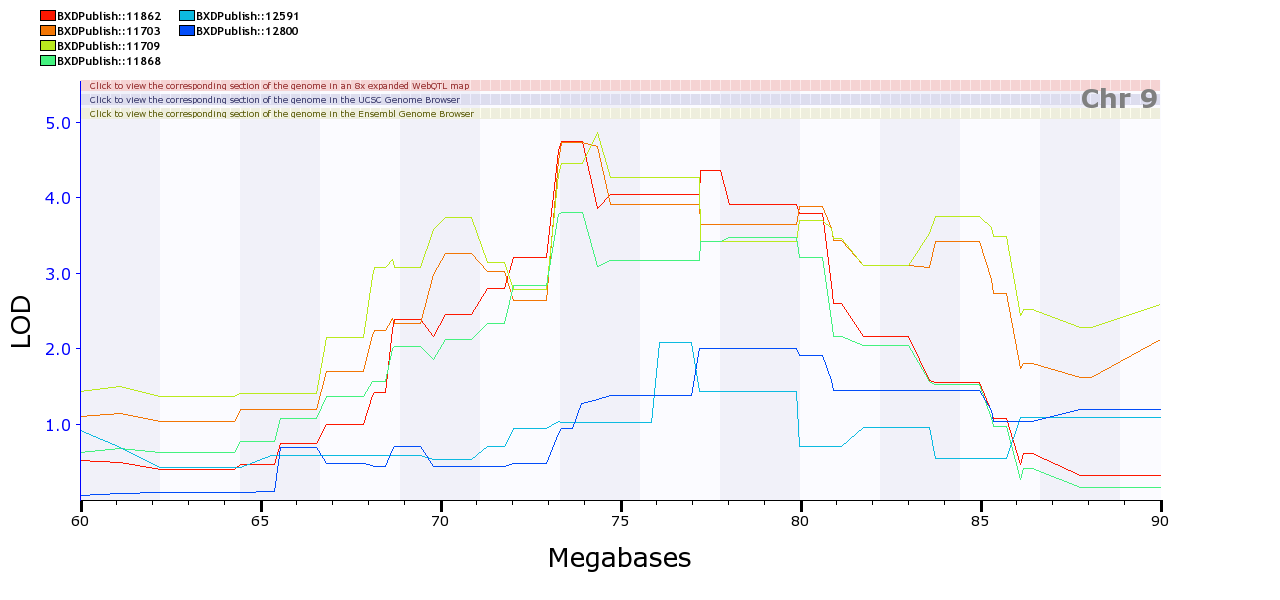

Supplement: S1 Fig — Genetic mapping results are shown for traits from the BXD Published Phenotypes Database. Association strength (LOD) is shown on the Y-axis and plotted over a 30 Mb region spanning the Gsta4 cis eQTL. Line colors show results of interval mapping for each trait plotted on the same scale according to the legend (upper left corner). Homovanillic acid levels (record ID = 12801, LOD = 2.7) and 5-hydroxyindoleacetic acid levels (record ID = 12800, LOD = 2) in the medial septal nucleus, locomotion in the open field periphery (record ID = 11862, LOD = 4.7), locomotion in the open field center (record ID = 11868, LOD = 3.8), locomotor activity after ethanol injection (record ID = 11703, LOD = 4.7), and volume of the hippocampus mossy fiber pathway (record ID = 12591, LOD = 2.1). (PNG) [file pone.0148230.s001.png]

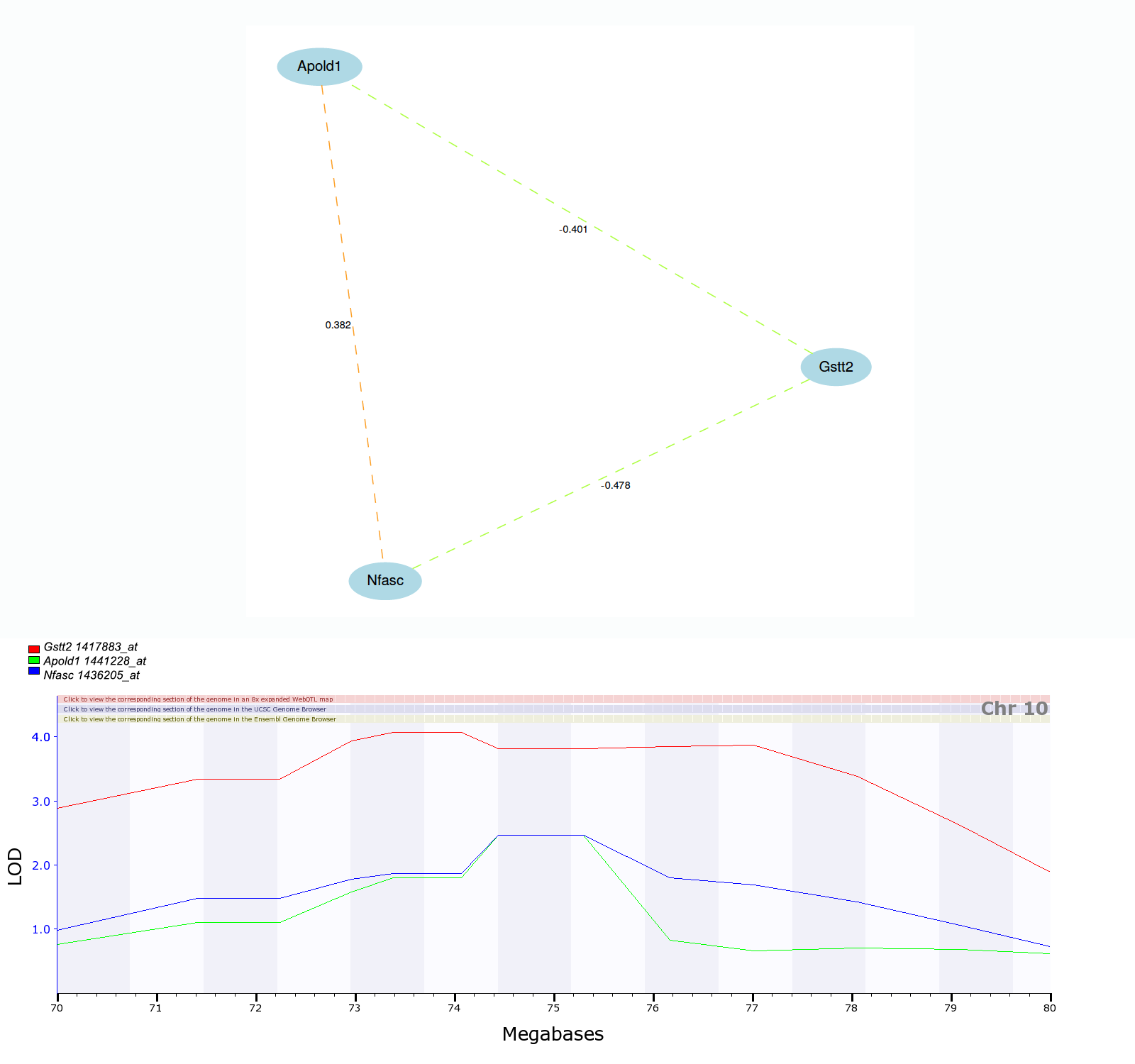

Supplement: S3 Fig — Top panel shows correlations between Gstt2 and transcripts mapping back to the Gstt2 locus. Bottom panel shows genetic mapping results for each transcript and Gstt2 (for reference, in red) in hippocampus. Association strength (LOD) is shown on the Y-axis and plotted over the region spanning the Gstt2 cis eQTL. Line colors show results of interval mapping for each trait plotted on the same scale according to the legend (upper left corner) in each panel. (TIF) [file pone.0148230.s003.tif]

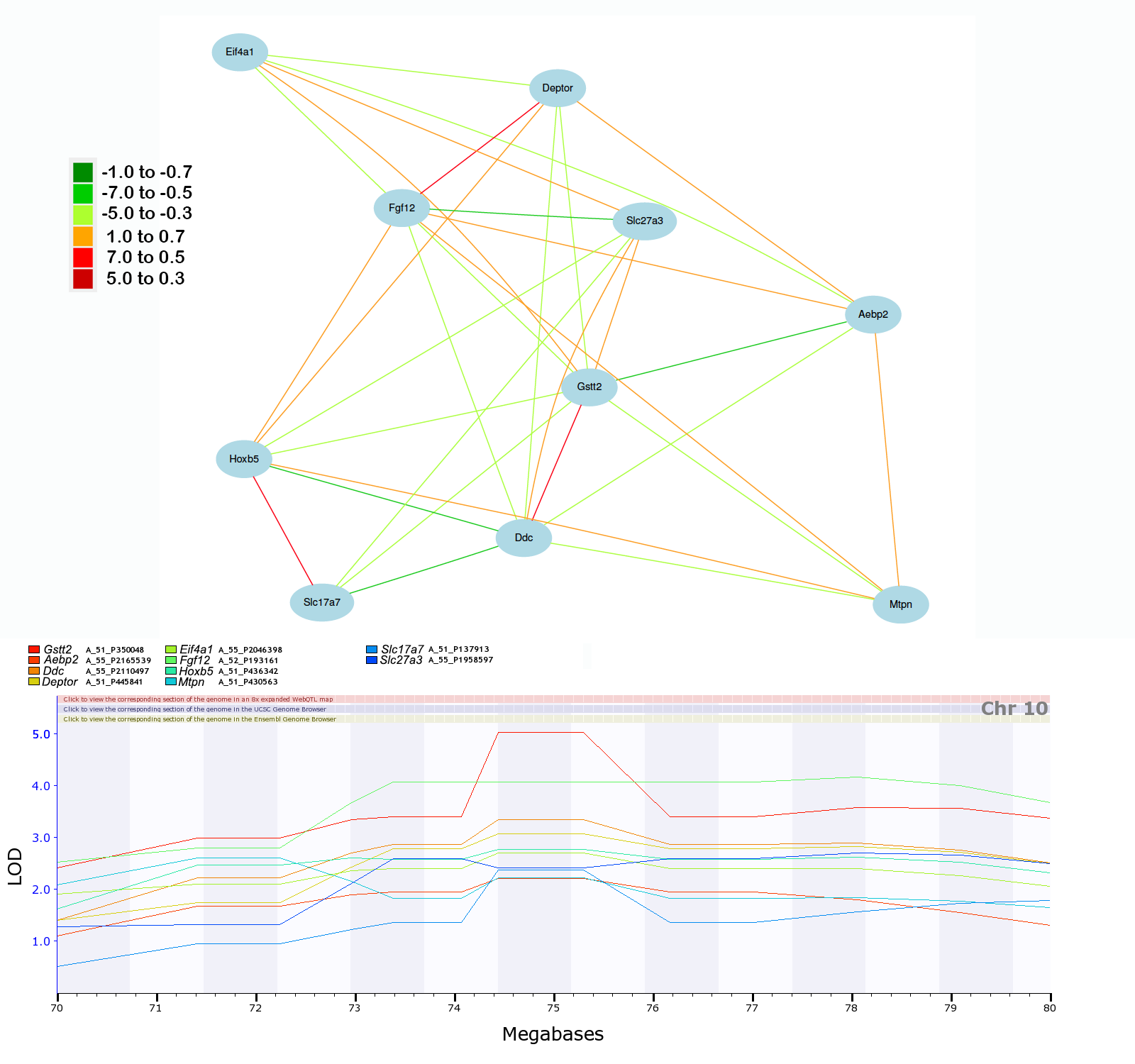

Supplement: S4 Fig — Top panel shows correlation network between Gstt2 and transcripts mapping back to the Gstt2 locus. Positive correlations are indicated by warm line (edge) colors and negative correlations are indicated by cool edge colors. Network threshold is r = |0.3|. Bottom panel shows genetic mapping results for each transcript and Gstt2 (for reference, in red) in midbrain. Association strength (LOD) is shown on the Y-axis and plotted over the region spanning the Gstt2 cis eQTL. Line colors show results of interval mapping for each trait plotted on the same scale according to the legend (upper left corner) in each panel. (TIF) [file pone.0148230.s004.tif]

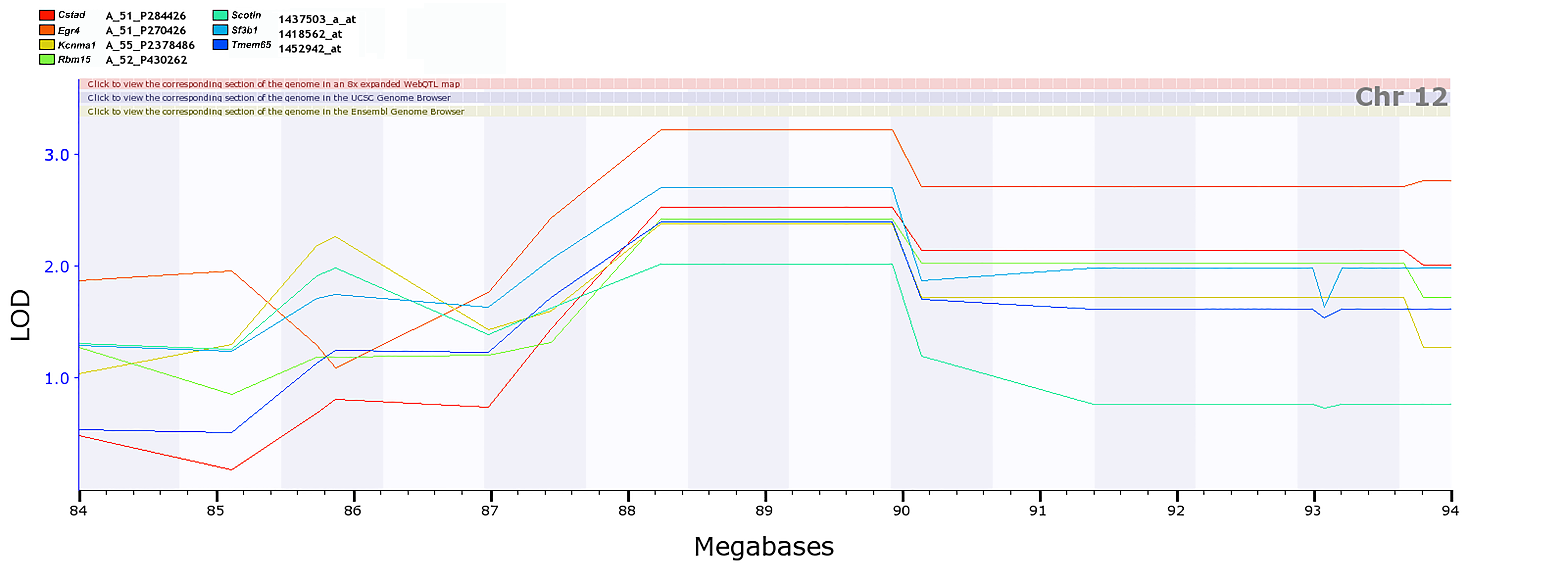

Supplement: S5 Fig — Genetic mapping results are shown for each transcript in hippocampus and midbrain. Association strength (LOD) is shown on the Y-axis and plotted over the region spanning the Gstz1 cis eQTL. Line colors show results of interval mapping for each trait plotted on the same scale according to the legend (upper left corner). (TIF) [file pone.0148230.s005.tif]

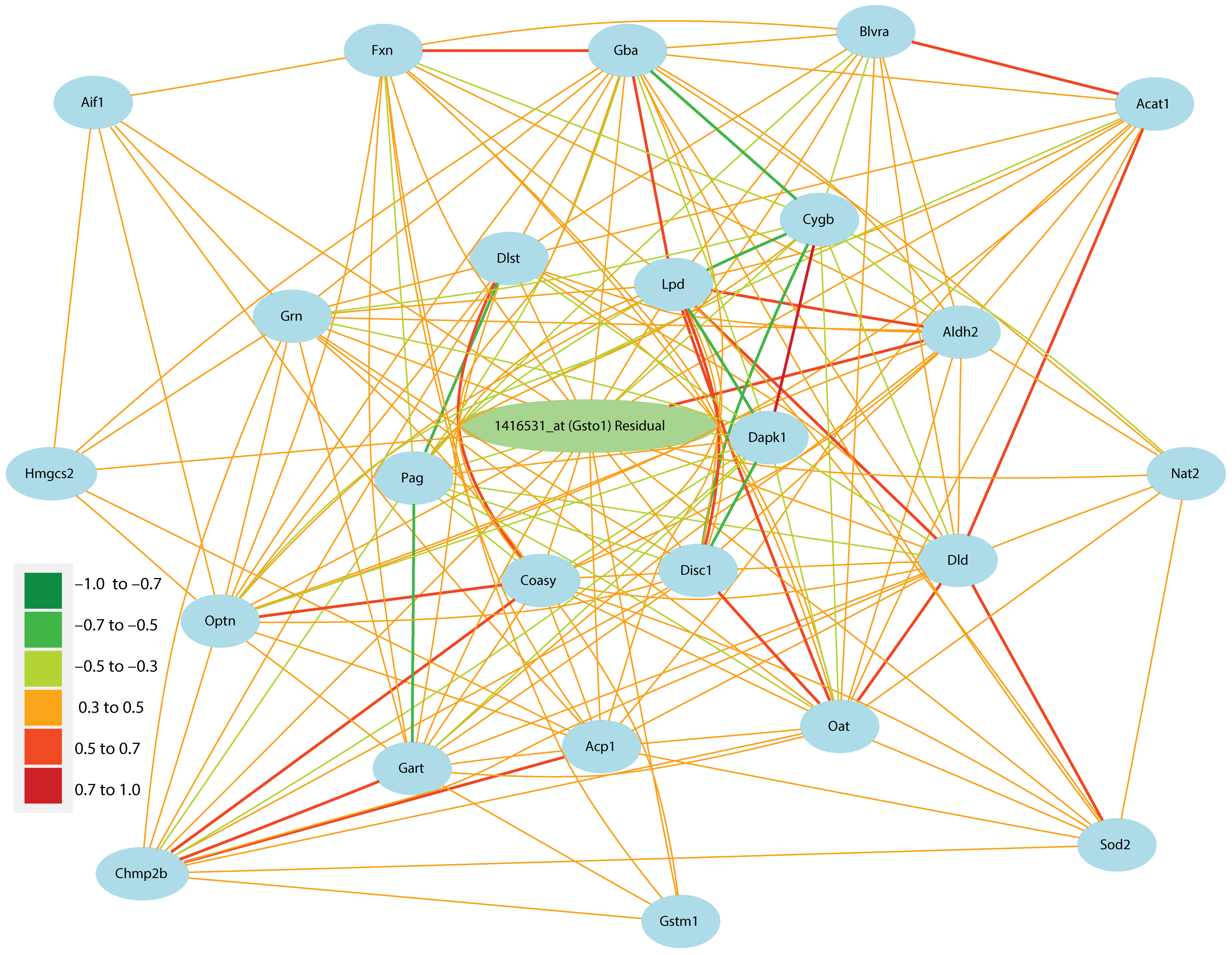

Supplement: S6 Fig — Network for the 25 AD associated genes that are correlated with hippocampal Gsto1 expression and co-cited in the literature. Positive correlations are indicated by warm line (edge) colors and negative correlations are indicated by cool edge colors. Network threshold is set at r = |0.3|. The center of the network is the Gsto1 residual trait generated after partial correlation analysis. All 25 genes are highly connected in this network. (TIF) [file pone.0148230.s006.tif]
